# Carbon quantum dots for sustainable energy: enhancing electrocatalytic reactions through structural innovation

**DOI:** 10.1039/d5na00205b

**Published:** 2025-05-07

**Authors:** Fadhel F. Sead, Yashwantsinh Jadeja, Anjan Kumar, Rekha M. M., Mayank Kundlas, Suman Saini, Kamal Kant Joshi, Hadi Noorizadeh

**Affiliations:** a Department of Dentistry, College of Dentistry, The Islamic University Najaf Iraq; b Department of Medical Analysis, Medical Laboratory Technique College, The Islamic University of Al Diwaniyah Al Diwaniyah Iraq; c Department of Medical Analysis, Medical Laboratory Technique College, The Islamic University of Babylon Babylon Iraq; d Marwadi University Research Center, Department of Chemistry, Faculty of Science, Marwadi University Rajkot-360003 Gujarat India; e Department of Electronics and Communication Engineering, GLA University Mathura-281406 India; f Department of Chemistry and Biochemistry, School of Sciences, JAIN (Deemed to be University) Bangalore Karnataka India; g Centre for Research Impact & Outcome, Chitkara University Institute of Engineering and Technology, Chitkara University Rajpura Punjab 140401 India; h Department of Chemistry, Chandigarh Engineering College, Chandigarh Group of Colleges-Jhanjeri Mohali 140307 Punjab India; i Department of Allied Science, Graphic Era Hill University Dehradun-248002 Uttarakhand India; j Graphic Era Deemed to be University Dehradun Uttarakhand India; k Department of Chemistry, Islamic Azad University Tehran Iran hadinoorizadeh@yahoo.com

## Abstract

Carbon quantum dots (CQDs) have emerged as a promising class of nanomaterials due to their unique optical, electrical, and catalytic properties, positioning them as key players in electrocatalytic applications. This review provides a comprehensive and up-to-date analysis of CQDs, focusing on their electrocatalytic behavior in critical reactions such as the oxygen evolution reaction (OER), hydrogen evolution reaction (HER), oxygen reduction reaction (ORR), carbon dioxide reduction reaction (CO_2_RR), bifunctional catalysis and liquid fuel electrooxidation. Distinct from prior studies, this study highlights recent innovations in CQD synthesis, including heteroatom doping and defect engineering, and explores their structural properties—like absorbance, photoluminescence, and electroluminescence—that enhance catalytic performance. We elucidate the electrocatalytic mechanisms (*e.g.*, reactant adsorption, electron transfer, and intermediate stabilization) and address challenges such as low conductivity and scalability, proposing advanced strategies like hybridization with transition metals. Additionally, this review uniquely emphasizes the potential of CQDs in bifunctional catalysis and environmental applications, offering fresh insights into their role in advancing sustainable energy technologies.

## Abstract 2

A comprehensive review highlights the electrocatalytic roles of carbon quantum dots in key energy-related reactions including the OER, the HER, the ORR, the CO_2_RR, liquid fuel electrooxidation, and bifunctional catalysis.

## Graphical abstract

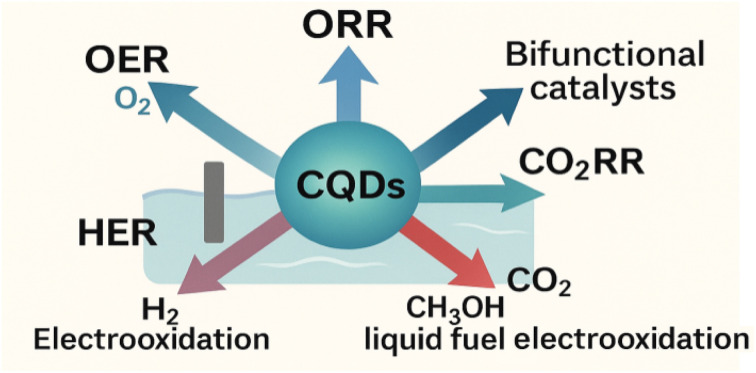

## 1. Introduction

Carbon quantum dots (CQDs) are an emerging class of carbon-based nanomaterials that have attracted significant attention due to their remarkable optical, electrical, and electrochemical properties. These nanomaterials, typically less than 10 nm in size, exhibit quantum confinement effects and unique surface chemistry, leading to tunable photoluminescence, high surface area, and excellent biocompatibility. A notable subclass of CQDs, graphene quantum dots (GQDs), has a two-dimensional (2D) graphene-like structure, characterized by a planar arrangement of sp^2^-hybridized carbon atoms with abundant edge sites. GQDs retain the quantum confinement properties of CQDs while offering enhanced electrical conductivity and structural stability, making them particularly promising for electrocatalytic and optoelectronic applications. In recent years, both CQDs and GQDs have found a broad range of applications, from bioimaging to energy storage and conversion.^1–3^ Among them, their role in electrocatalysis—particularly in energy conversion reactions such as the oxygen evolution reaction (OER), hydrogen evolution reaction (HER), and carbon dioxide reduction (CO_2_RR)—has been increasingly explored.^4,5^ The growing need for sustainable energy has highlighted the importance of developing efficient, low-cost, and durable electrocatalysts. Traditional electrocatalysts such as platinum and iridium oxide are expensive and scarce, limiting their practical application in large-scale energy systems. As a result, CQDs and GQDs have emerged as alternative catalyst materials due to their abundant availability, ease of synthesis, and the ability to modify their electronic properties to suit various catalytic processes. CQDs and GQDs have the potential to outperform conventional catalysts, offering high efficiency, low cost, and enhanced long-term stability.^6,7^

The unique electronic properties of CQDs, combined with their ability to incorporate heteroatoms (*e.g.*, nitrogen, sulfur, and phosphorus), enable significant improvements in electrocatalytic performance. By doping with heteroatoms, CQDs can enhance charge transfer, reduce activation energy, and increase the number of active sites on their surface, thereby optimizing their performance in key reactions like the OER, HER, and CO_2_RR. Moreover, CQDs can be integrated with metals or metal oxides to form hybrid systems that demonstrate synergistic effects, further improving catalytic efficiency.^4,8^ In terms of synthesis, several methods are employed to produce CQDs, each influencing the final properties of the material. These include top-down methods such as laser ablation, arc discharge, and electrochemical oxidation, which break down larger carbon sources into nanoscale materials. Bottom-up methods like hydrothermal and solvothermal synthesis, as well as microwave-assisted synthesis, offer more control over particle size and surface functionalization, yielding CQDs with tailored properties. Additionally, green synthesis techniques, including biomass-derived and enzymatic methods, have gained attention for their eco-friendliness and cost-effectiveness.^8,9^

In the field of electrocatalysis, CQDs have shown promise in various reactions, particularly in energy conversion processes like the OER and HER. The OER, essential for water splitting to generate oxygen, typically suffers from high overpotentials and slow kinetics. CQDs, especially when integrated with transition metals or perovskite materials, have demonstrated enhanced catalytic activity, reducing overpotentials and improving efficiency. Similarly, CQDs have been explored for the HER, which involves proton reduction to form hydrogen gas. The incorporation of CQDs into metal-based catalysts has improved HER performance; achieving comparable results to noble metal-based systems. CQDs have also been investigated for their role in the ORR, which is critical for fuel cell applications. When coupled with carbon-based materials like graphene or carbon nanotubes, CQDs exhibit excellent ORR activity, enhancing electron transfer and reducing catalyst degradation. Furthermore, CQDs are being studied for their ability to catalyse the CO_2_RR, a process with significant potential for carbon capture and utilization. By incorporating CQDs into metal catalysts, researchers have achieved enhanced CO_2_RR efficiency, demonstrating their potential in addressing global environmental challenges.^10–12^

Beyond these primary applications, CQDs also show promise in liquid fuel electrooxidation and as bifunctional catalysts. In liquid fuel electrooxidation, CQDs help accelerate the oxidation of fuels like methanol and ethanol, providing an eco-friendly and efficient approach to energy conversion. Additionally, bifunctional catalysts that can perform multiple reactions, such as the OER and HER, in a single system, benefit from the incorporation of CQDs due to their versatile properties and ability to enhance reaction kinetics for both reactions.^12,13^ CQDs are proving to be a highly versatile material in the field of electrocatalysis, offering a promising alternative to traditional catalysts. Their unique properties, combined with advances in synthesis methods and surface functionalization, have positioned them as a powerful tool in energy conversion systems. Ongoing research continues to focus on overcoming challenges related to their synthesis, stability, and scalability, with the aim of unlocking their full potential in sustainable energy applications.^14,15^ While prior reviews have broadly surveyed the properties and applications of CQDs, this work sets itself apart by delivering an in-depth exploration of the latest synthesis advancements—such as heteroatom doping, defect engineering, and single-step electrodeposition—and their impact on electrocatalytic performance. We provide a rigorous analysis of electrocatalytic mechanisms, including quantitative insights into charge transfer kinetics and intermediate stabilization, which are often underexplored in earlier studies. Moreover, this review uniquely bridges the gap between fundamental research and practical applications by evaluating the emerging roles of CQDs in bifunctional electrocatalysis (*e.g.*, OER/HER systems) and environmental remediation, areas witnessing rapid progress since 2020. These distinctions position this review as a critical resource for understanding the evolving landscape of CQD-based electrocatalysts in sustainable energy technologies.

## 2. Properties of carbon quantum dots

### 2.1. Absorbance

**Fig. 1:**
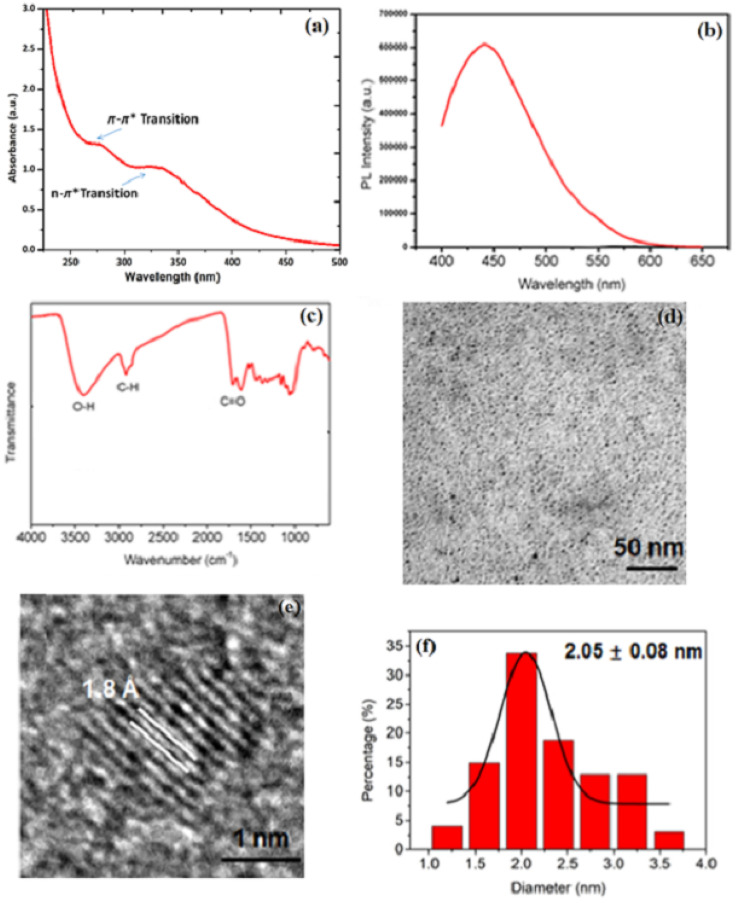
(a) Structural characterization of CQDs. (b) The photoluminescence (PL) spectra of CQDs at 10 μg mL^−1^ under natural light irradiation. (c) The FTIR spectra of CQDs. Morphological characterization of CQDs. Low and high-resolution TEM images and particle size distributions of (d–f) CQDs. Reproduced from Fu *et al.* (2023),^16^*Heliyon*, under the terms of the Creative Commons Attribution License (CC BY 4.0).

CQDs predominantly exhibit optical absorption in the ultraviolet range, extending into the visible spectrum. The primary optical absorption peaks in the ultraviolet-visible region are attributed to transitions involving sp^2^ carbon to π–π and the hybridization change of n–π with a heteroatom. These transitions provide valuable information about the chemical structure, bond types, and possible functional groups present in the material, while surface passivation or modification can significantly influence these absorption properties. CQDs possess the ability to absorb a broad spectrum of photons. When integrated into electrodes or electrolytes, the absorbed light energy facilitates charge separation within the material. Additionally, light absorption by CQDs in the electrolyte can alter its pH, viscosity, and ion mobility, thereby optimizing the electrochemical performance under various operational conditions (Fig. 1a).^16^

### 2.2. Photoluminescence

The photoluminescence (PL) spectrum reveals how CQDs interact with light, influencing their emission characteristics and potential applications. The peak appears to be in the range of approximately 450–500 nm, rather than around 460 nm (in the visible blue region), suggesting that upon excitation, CQDs emit blue light, a behavior common in quantum dots and fluorescent nanomaterials with discrete energy levels. This intrinsic PL arises from quantum confinement effects, surface defects, and conjugated π-domains, which can be tuned through doping, functionalization, or structural modifications. Beyond optoelectronic applications such as LEDs and bioimaging, the PL properties of CQDs play a crucial role in electrocatalysis by modulating charge transfer dynamics and electronic interactions at the electrode interface. Their ability to absorb and re-emit photons facilitates photo-induced electron transfer processes, enhancing catalytic performance in reactions like the ORR and HER. Furthermore, the presence of PL-active functional groups and heteroatom doping (*e.g.*, nitrogen, sulfur, or phosphorus) influences fluorescence properties and catalytic behavior, improving charge transfer kinetics. Additionally, the tunability of PL characteristics allows real-time monitoring of electrochemical reactions by tracking fluorescence intensity variations, offering valuable insights into catalytic activity and reaction mechanisms.^17,18^ The synergy between PL and electrocatalysis highlights the multifunctionality of CQDs, making them promising materials for energy conversion, sensing, and advanced electrochemical applications, as demonstrated in Fig. 1b.

### 2.3. Electroluminescence

Electroluminescence (EL) in the electrocatalytic behavior of CQDs is recognized as an innovative feature with significant potential for enhancing the efficiency of electrochemical reactions. CQDs, carbon-based nanoparticles with nanometer-scale dimensions, have gained considerable attention due to their distinctive optical properties, including electroluminescence in electrochemical reactions. This phenomenon arises from the quantum confinement effect, which creates discrete energy levels within the CQD structure. When an external voltage is applied, electrons and holes are injected into the CQDs, where they recombine, resulting in light emission. The characteristics of this light emission depend on the size, surface functionalization, and surrounding environment of the CQDs, making them ideal candidates for various applications in electrocatalysis. In electrocatalysis, the electroluminescent properties of CQDs can be utilized to monitor and improve catalytic processes such as the ORR, HER, and CO_2_RR. The light emission during electrochemical reactions serves as an indicator of catalytic activity, allowing real-time monitoring of reaction dynamics. Moreover, the electroluminescent behavior of CQDs can facilitate charge transfer processes, enhancing the efficiency and selectivity of electrocatalytic reactions.^18,19^ The interaction between CQDs and the electrochemical environment enables the tuning of their electroluminescent properties, positioning them as promising candidates for next-generation electrocatalytic materials in energy conversion and storage applications.

### 2.4. Structural, optical, and morphological characterization of CQDs

To comprehensively understand the structural, optical, and morphological characteristics of the synthesized CQDs, various spectroscopic and microscopic techniques were employed. These analyses provide critical insights into the chemical composition, surface functionalization, particle size distribution, and crystalline nature of the CQDs. The following sections describe the results obtained from UV-vis absorption spectroscopy, photoluminescence (PL) spectroscopy, FTIR spectroscopy, TEM, high-resolution TEM (HRTEM), and particle size distribution analysis, each contributing to a deeper understanding of the material's properties. Fig. 1c provides an FTIR spectrum, which is crucial for identifying functional groups based on molecular vibrations. The broad peak at 3400 cm^−1^ corresponds to O–H stretching, suggesting the presence of hydroxyl groups, which could be due to surface modification or oxidation of the material. The C–H stretching at around 2900 cm^−1^ is indicative of aliphatic chains, possibly suggesting organic capping agents or residual organic solvents. The sharp C=O peak at 1700 cm^−1^ is characteristic of carbonyl groups, indicating oxidation or functionalization with oxygen-containing groups. The presence of these functional groups plays a significant role in CQD solubility, stability, and interaction with other substances, particularly in aqueous and biological environments. Functionalization with oxygen-containing groups can enhance the hydrophilicity of CQDs, making them more dispersible in polar solvents and increasing their applicability in bioimaging and drug delivery.

In Fig. 1d, the TEM image provides a nanoscale visualization of CQD morphology. The image, with a scale bar of 50 nm, reveals that the material consists of small, spherical particles with a relatively uniform size distribution. Such morphology is characteristic of well-synthesized CQDs, where the absence of large agglomerates suggests that the synthesis process effectively prevents uncontrolled particle growth and aggregation. The small size and spherical nature of CQDs are essential for applications where high surface area and quantum confinement effects play a role, such as in catalysis, biosensing, and energy storage devices. Fig. 1e shows a higher-magnification HRTEM image, offering atomic-level insights into the structural organization of the CQDs. The visible lattice fringes with an interplanar distance of 1.8 Å confirm the crystalline nature of the material. This spacing corresponds to the graphitic (002) plane, indicating that the CQDs retain a degree of graphitic ordering despite their nanoscale size. The crystallinity of CQDs plays a crucial role in determining their electronic properties, as a well-defined lattice structure enhances charge transport, conductivity, and optical behavior. Understanding the atomic arrangement is particularly important for applications in optoelectronics and photovoltaic devices, where controlled crystallinity can optimize the efficiency of charge carrier dynamics.

Fig. 1f presents a histogram of the particle size distribution, obtained through a statistical analysis of the TEM images. The data indicate an average particle size of 2.05 ± 0.08 nm, with a relatively narrow distribution. Maintaining uniform particle size is critical, as many physicochemical properties, such as fluorescence quantum yield, catalytic activity, and charge transfer efficiency, are strongly size-dependent. The narrow distribution suggests that the synthesis method used is highly effective and reproducible, ensuring consistency in CQD properties. Such control over size distribution is beneficial in applications where uniformity directly influences performance. By integrating FTIR, TEM, HRTEM, and size distribution analysis, this characterization provides a comprehensive understanding of CQD composition, structure, and morphology. The presence of oxygen-containing functional groups, as evidenced by FTIR, indicates potential for surface modification and enhanced solubility. TEM and HRTEM images confirm the small size, spherical shape, and crystallinity, which are essential for applications requiring quantum confinement effects and high surface reactivity. The size distribution data further validate the uniformity of the particles, reinforcing the precision of the synthesis method. Together, these findings highlight the high-quality synthesis of CQDs and their suitability for a broad range of applications.^16–19^

The surface chemistry of CQDs plays a pivotal role in determining their electrocatalytic activity, solubility, and interaction with reactants, making surface-sensitive analytical techniques essential for complete characterization. X-ray photoelectron spectroscopy (XPS) is widely employed to probe the elemental composition and chemical states of CQD surfaces, offering quantitative insights into the presence of carbon, oxygen, nitrogen, and other heteroatoms, as well as their bonding environments. For instance, XPS can distinguish between sp^2^ and sp^3^ carbon (C 1s peaks at ∼284.5 eV and ∼285.5 eV, respectively), identify oxygen-containing functional groups (*e.g.*, C=O at ∼531–532 eV in O 1s spectra), and confirm heteroatom doping (*e.g.*, pyridinic, pyrrolic, and graphitic nitrogen at ∼398.5 eV, ∼400.5 eV, and ∼401.5 eV in N 1s spectra). Such information is critical for understanding how surface modifications, like doping or functionalization, enhance charge transfer and catalytic performance in reactions such as the OER and HER. A study by Nallayagari *et al.*^20^ utilized XPS to characterize boron- and nitrogen-co-doped CQDs, revealing how co-doping alters the electronic structure and boosts ORR activity, underscoring the technique's value in electrocatalyst development.

Complementary surface-sensitive methods, such as Auger electron spectroscopy (AES) and time-of-flight secondary ion mass spectrometry (ToF-SIMS), can further elucidate surface properties. AES provides high spatial resolution for mapping elemental distributions, while ToF-SIMS offers molecular-level insights into surface functional groups and their spatial arrangement. Together, these techniques enable a comprehensive understanding of the surface chemistry of CQDs, bridging the gap between structural features and electrocatalytic functionality. For example, XPS combined with TEM and FTIR has been used to correlate surface oxygen content with photoluminescence behavior, highlighting the interplay between surface states and optical properties.^19^ By integrating surface analysis with other characterization methods, researchers can tailor the surface properties of CQDs for specific applications, enhancing their efficacy in sustainable energy technologies.

## 3. Synthesis methods for CQDs

### 3.1. Top-down methods

#### 3.1.1. Laser ablation

Laser ablation of CQDs involves using pulsed lasers, typically with wavelengths in the range of 1064 nm (Nd:YAG lasers) or 532 nm, to irradiate a carbon precursor. The energy per pulse is often in the range of 100 mJ to 1 J, with pulse durations between nanoseconds (ns) and femtoseconds (fs), depending on the desired effect. For example, shorter pulse durations (femtosecond lasers) typically lead to more uniform CQD sizes and fewer defects compared to longer pulse durations (nanoseconds). The ablation process can occur in liquids such as water, ethanol, or other solvents, which helps in cooling the plasma and promoting CQD formation. In one study, laser ablation of graphite in water with a 1064 nm, 100 mJ pulsed laser produced CQDs with an average diameter of 5 nm and a strong blue fluorescence emission under UV light (365 nm).^21^

Laser ablation offers tunability by adjusting the laser fluence, which is the energy delivered per unit area. For example, increasing the fluence from 10 J cm^−2^ to 20 J cm^−2^ has been shown to reduce the size of CQDs from ∼10 nm to ∼3 nm, enhancing their quantum confinement effect and altering their photoluminescence. The ablation time also affects the final CQD size and yield. For instance, longer irradiation times lead to smaller CQD sizes but may reduce overall yield due to excessive fragmentation. In one experiment, increasing the laser ablation time from 10 minutes to 30 minutes reduced the average CQD size from 7 nm to 3.5 nm, but the yield dropped by 20%. Despite its precision, the yield of CQDs through laser ablation is typically lower than that with other synthesis methods. Yields can range from 5 to 15 mg of CQDs per gram of carbon precursor, depending on parameters such as laser power and ablation duration. While this low yield can limit large-scale applications, laser ablation remains advantageous for producing CQDs with high purity and well-controlled optical properties. Furthermore, the absence of harsh chemical reagents in this method makes it suitable for producing CQDs intended for biological applications, such as bioimaging or drug delivery, where purity and non-toxicity are critical.^22^

#### 3.1.2. Arc discharge

The arc discharge method is a classic and efficient technique for synthesizing carbon-based nanomaterials, including CQDs. This method involves creating an electric arc between two carbon electrodes, typically made of graphite, in a controlled atmosphere. When a high current (50–150 A) and low voltage (10–30 V) are applied, the intense heat generated by the arc (reaching temperatures of 3000–4000 °C) vaporizes the carbon from the anode. The vaporized carbon atoms then condense in the cooler regions of the chamber, forming CQDs along with other carbon nanostructures like fullerenes, nanotubes, and graphene. The process is usually carried out in an inert gas atmosphere (*e.g.*, argon or helium) to prevent oxidation and control the size and morphology of the resulting nanoparticles. The size and properties of the CQDs produced by arc discharge depend on several factors, including the current density, gas pressure, and type of gas used. For example, introducing hydrogen into the inert gas mixture can help reduce the size of the CQDs and improve their fluorescence properties.^23^ Studies have shown that CQDs synthesized *via* arc discharge typically range in size from 2 to 10 nm, with quantum yields (QY) varying between 5% and 20%, depending on the synthesis conditions and post-treatment processes.

The method is known for its high yield and ability to produce CQDs with good crystallinity and tunable optical properties. However, the process often requires post-synthesis purification to separate CQDs from other carbon byproducts like soot or graphite particles. Despite its advantages, the arc discharge method has some limitations. The high energy consumption, need for specialized equipment, and generation of mixed carbon byproducts make it less cost-effective and scalable compared to other methods like hydrothermal or microwave-assisted synthesis. However, it remains a valuable technique for producing high-quality CQDs with unique properties, particularly for applications in optoelectronics, catalysis, and bioimaging. Recent advancements have focused on optimizing the arc discharge process by using alternative carbon sources (*e.g.*, biomass-derived carbon) or combining it with other techniques to enhance the yield and functionality of CQDs.^24^

#### 3.1.3. Electrochemical oxidation

Electrochemical oxidation is a widely used method for synthesizing CQDs due to its simplicity, cost-effectiveness, and ability to produce high-quality CQDs with tunable properties. This method involves the electrochemical oxidation of carbon-based materials, such as graphite, carbon fibers, or carbon nanotubes, in an electrolyte solution. When a voltage is applied, the carbon material undergoes oxidation, leading to the formation of small carbon fragments that eventually form CQDs. The process is typically carried out in an electrochemical cell with a carbon-based working electrode, a counter electrode (*e.g.*, platinum), and a reference electrode (*e.g.*, Ag/AgCl). The electrolyte solution often contains water or organic solvents with supporting electrolytes like NaOH, KOH, or H_2_SO_4_, which facilitate the oxidation process.^25^

The size, morphology, and optical properties of the synthesized CQDs can be controlled by adjusting various parameters, such as the applied voltage, electrolyte composition, and reaction time. For example, studies have shown that applying a voltage of 2–5 V for 1–10 hours can yield CQDs with sizes ranging from 2 to 10 nm and photoluminescence (PL) emission wavelengths between 400 and 600 nm. The electrochemical oxidation process also allows for the introduction of functional groups (*e.g.*, carboxyl and hydroxyl) on the surface of the CQDs, which enhances their solubility, stability, and potential applications in sensing, bioimaging, and catalysis. Additionally, this method is environmentally friendly, as it avoids the use of harsh chemicals and high temperatures, making it a sustainable approach for CQD synthesis. Recent advancements in electrochemical oxidation have focused on improving the yield and quality of CQDs. For instance, researchers have demonstrated that using ionic liquids as electrolytes can enhance the efficiency of the oxidation process and produce CQDs with higher quantum yields (up to 20–30%). Moreover, the use of renewable carbon sources, such as biomass-derived carbon or waste materials, has been explored to make the process more sustainable. For example, CQDs synthesized from coffee grounds or banana peels *via* electrochemical oxidation have shown excellent PL properties and biocompatibility, making them suitable for biomedical applications. Overall, electrochemical oxidation is a versatile and scalable method for synthesizing CQDs with tailored properties, offering significant potential for various technological and industrial applications.^26^

#### 3.1.4. Ultrasonic treatment

Ultrasonic treatment is a simple, efficient, and eco-friendly method for synthesizing CQDs. This technique utilizes high-frequency ultrasonic waves (typically 20–40 kHz) to break down carbon precursors into nanoscale particles. The process involves the phenomenon of acoustic cavitation, where the formation, growth, and collapse of microbubbles in a liquid medium generate extreme local temperatures (up to 5000 K) and pressures (up to 1000 atm). These conditions facilitate the decomposition of carbon precursors and the formation of CQDs. Common precursors include citric acid, glucose, and biomass, which are dissolved in water or other solvents and subjected to ultrasonic irradiation for a specific duration (*e.g.*, 1–6 hours). The method is highly versatile, allowing for the control of CQD size, surface functionalization, and optical properties by adjusting parameters such as ultrasonic power, frequency, and reaction time.^27^

One of the key advantages of ultrasonic treatment is its ability to produce CQDs under mild conditions without the need for high temperatures, strong acids, or complex equipment. For example, studies have shown that ultrasonic treatment of citric acid at 40 kHz for 3 hours can yield CQDs with a size range of 2–6 nm and strong blue fluorescence under UV light. The method also allows for the incorporation of heteroatoms (*e.g.*, nitrogen and sulfur) into the CQD structure by using nitrogen- or sulfur-containing precursors, which can enhance the quantum yield and catalytic properties of the CQDs. Additionally, ultrasonic treatment is scalable and can be used to synthesize CQDs in large quantities, making it suitable for industrial applications. Despite its advantages, ultrasonic treatment has some limitations. The yield of CQDs can be lower compared to that in other methods like hydrothermal synthesis, and the process may require post-treatment purification to remove unreacted precursors or byproducts. However, the method's simplicity, cost-effectiveness, and ability to produce high-quality CQDs with tunable properties make it a popular choice for researchers.^28^

#### 3.1.5. Chemical oxidation

Chemical oxidation is a widely used method for synthesizing CQDs. This approach involves the oxidation of carbon-rich materials, such as graphite, carbon nanotubes, or coal, using strong oxidizing agents like HNO_3_, H_2_SO_4_, or a mixture of both. The process breaks down the larger carbon structures into smaller fragments, which are then functionalized with oxygen-containing groups (*e.g.*, carboxyl, hydroxyl, and carbonyl groups) on their surface. These functional groups not only stabilize the CQDs but also enhance their solubility in water and other polar solvents, making them suitable for various applications such as bioimaging, sensing, and catalysis.^29^

**Fig. 2:**
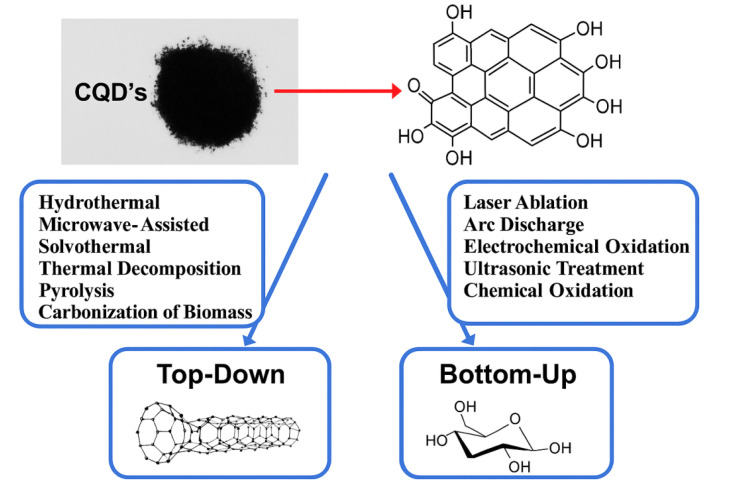
The synthesis methods of CQDs, comparing the top-down and bottom-up approaches.

The synthesis typically involves refluxing the carbon precursor in a concentrated acid solution at elevated temperatures (*e.g.*, 80–120 °C) for several hours. For example, graphite powder can be oxidized using a mixture of HNO_3_ and H_2_SO_4_ (1 : 3 ratio) at 120 °C for 24 hours. During this process, the acid mixture intercalates into the graphite layers, breaking the sp^2^ carbon network and introducing oxygen-containing functional groups. The resulting oxidized material is then subjected to further processing, such as centrifugation, dialysis, or filtration, to isolate the CQDs. The size of the CQDs can be controlled by adjusting the reaction time, temperature, and the concentration of the oxidizing agents. Studies have shown that CQDs synthesized *via* chemical oxidation typically range in size from 2 to 10 nm, with a quantum yield (QY) of 5–20%, depending on the precursor and reaction conditions. One of the key advantages of the chemical oxidation method is its simplicity and cost-effectiveness, as it does not require expensive equipment or complex procedures. However, the use of harsh chemicals and the need for extensive purification to remove residual acids and byproducts are significant drawbacks. Additionally, the strong oxidation process can introduce defects in the carbon structure, which may affect the optical and electronic properties of the CQDs.^30^ Despite these challenges, chemical oxidation remains a popular method for synthesizing CQDs due to its scalability and ability to produce highly functionalized nanoparticles. Fig. 2 illustrates the synthesis methods of CQDs, comparing the top-down and bottom-up approaches.

### 3.2. Bottom-up methods

#### 3.2.1. Hydrothermal synthesis

Hydrothermal synthesis of CQDs is one of the most widely used and efficient methods for producing CQDs due to its simplicity, cost-effectiveness, and ability to control the size and properties of the resulting nanoparticles. In this method, carbon-rich precursors (*e.g.*, citric acid, glucose, or biomass) are dissolved in water or another solvent and placed in a sealed autoclave. The mixture is then heated to temperatures typically ranging from 120 °C to 250 °C for several hours (usually 2–12 hours), under autogenous pressure. During this process, the precursors undergo carbonization, dehydration, and polymerization, leading to the formation of CQDs. The size, fluorescence, and surface functionalization of the CQDs can be tuned by adjusting parameters such as temperature, reaction time, pH, and the type of precursor used. For example, citric acid is a popular precursor because it produces highly fluorescent CQDs with a quantum yield of up to 80% under optimized conditions.^31^

One of the key advantages of hydrothermal synthesis is its eco-friendliness, as it often uses water as the solvent and avoids toxic chemicals. Additionally, the method is highly versatile, allowing for the incorporation of heteroatoms (*e.g.*, nitrogen and sulfur) into the CQD structure by adding dopants like urea or thiourea during the reaction. This doping can enhance the optical and electronic properties of the CQDs, making them suitable for applications such as bioimaging, sensing, and photocatalysis. For instance, nitrogen-doped CQDs synthesized hydrothermally have shown improved fluorescence and catalytic activity compared to undoped CQDs. This method also enables the use of renewable and low-cost carbon sources, such as fruit peels, vegetable waste, or biomass, making it a sustainable approach for large-scale production. Despite its advantages, hydrothermal synthesis has some limitations. The process requires precise control over reaction conditions to achieve consistent results, and the yield of CQDs can vary depending on the precursor and reaction parameters. Additionally, the as-synthesized CQDs often require purification steps, such as dialysis or centrifugation, to remove unreacted precursors or byproducts. Nevertheless, hydrothermal synthesis remains a popular choice due to its scalability and ability to produce high-quality CQDs with tunable properties.^32^

#### 3.2.2. Solvothermal synthesis

Solvothermal synthesis of CQDs is a widely used bottom-up approach for producing CQDs due to its simplicity, scalability, and ability to control the size and surface properties of the resulting nanoparticles. In this method, carbon-rich precursors (*e.g.*, citric acid, glucose, or organic polymers) are dissolved in a solvent (water or organic solvents) and heated in a sealed autoclave at elevated temperatures (typically 120–250 °C) for several hours. The high temperature and pressure inside the autoclave facilitate the decomposition, carbonization, and nucleation of the precursors, leading to the formation of CQDs. The size, fluorescence, and surface functionalization of the CQDs can be tuned by varying parameters such as the precursor type, solvent, reaction temperature, and duration.^33^

One of the key advantages of solvothermal synthesis is its versatility in using a wide range of carbon precursors and solvents. For example, citric acid is a popular precursor due to its ability to produce highly fluorescent CQDs, while glucose and other carbohydrates are often used due to their low cost and abundance. The choice of solvent (*e.g.*, water, ethanol, or dimethylformamide) also plays a critical role in determining the properties of the CQDs. For instance, water-based solvothermal synthesis typically yields hydrophilic CQDs with oxygen-containing functional groups, making them suitable for biological applications. In contrast, organic solvents can produce hydrophobic CQDs with tailored surface properties for use in energy storage or catalysis. Studies have shown that reaction temperatures between 160 °C and 200 °C for 4–12 hours are optimal for producing CQDs with sizes ranging from 2 to 10 nm and strong photoluminescence.^34^

The solvothermal method is also known for its high yield and ability to produce CQDs with excellent optical properties. For example, CQDs synthesized from citric acid and ethylenediamine *via* solvothermal methods exhibit quantum yields (QY) of up to 80%, making them highly suitable for applications in bioimaging and sensing. Additionally, the method allows for the incorporation of heteroatoms (*e.g.*, nitrogen and sulfur) during synthesis, which can enhance the electronic and catalytic properties of the CQDs. Despite its advantages, the solvothermal method requires precise control over reaction conditions to avoid the formation of by-products or irregular-sized particles. Overall, solvothermal synthesis is a robust and efficient technique for producing high-quality CQDs with tunable properties for diverse applications.^33,34^

#### 3.2.3. Microwave-assisted synthesis

Microwave-assisted synthesis of CQDs is a rapid, efficient, and scalable method for producing CQDs. This technique utilizes microwave irradiation to heat carbon precursors, leading to the formation of CQDs through carbonization and surface functionalization. The process typically involves dissolving carbon-rich precursors (*e.g.*, citric acid, glucose, or urea) in water or another solvent and subjecting the mixture to microwave radiation for a few minutes. The microwave energy causes rapid and uniform heating, which accelerates the decomposition and carbonization of the precursors, resulting in the formation of CQDs. This method is highly tunable, as the size, fluorescence, and surface properties of the CQDs can be controlled by adjusting parameters such as microwave power, irradiation time, and precursor concentration.^35^

One of the key advantages of microwave-assisted synthesis is its speed. For example, CQDs can be synthesized in as little as 2–10 minutes, compared to hours or days required for hydrothermal or solvothermal methods. Additionally, the process is energy-efficient and environmentally friendly, as it often uses water as a solvent and avoids the need for harsh chemicals. Studies have shown that CQDs synthesized *via* microwave irradiation exhibit strong photoluminescence, with quantum yields (QY) ranging from 10% to 80%, depending on the precursor and synthesis conditions. For instance, citric acid and urea are commonly used precursors, with citric acid-derived CQDs often exhibiting blue fluorescence and urea-derived CQDs showing tunable emission across the visible spectrum. Despite its advantages, microwave-assisted synthesis has some limitations. The rapid heating can sometimes lead to inhomogeneous particle sizes or aggregation if not carefully controlled. However, these challenges can be mitigated by optimizing reaction conditions, such as using pulsed microwave irradiation or adding stabilizing agents. Overall, microwave-assisted synthesis is a versatile and efficient method for producing high-quality CQDs, making it suitable for applications in bioimaging, sensing, catalysis, and energy storage.^36^

#### 3.2.4. Pyrolysis

Pyrolysis is a widely used method for synthesizing CQDs due to its simplicity, scalability, and ability to produce CQDs with tunable properties. In this process, carbon-rich precursors, such as citric acid, glucose, urea, or biomass, are heated at high temperatures (typically between 300 °C and 600 °C) in an inert atmosphere (*e.g.*, nitrogen or argon) to induce thermal decomposition. During pyrolysis, the organic precursors undergo carbonization, breaking down into smaller carbon structures that eventually form CQDs. The size, morphology, and surface properties of the resulting CQDs can be controlled by adjusting parameters such as temperature, heating rate, and precursor composition. For example, citric acid is a common precursor that yields highly fluorescent CQDs when pyrolyzed at around 200–300 °C, while higher temperatures (400–600 °C) are often used for biomass-derived CQDs.^37^

One of the key advantages of pyrolysis is its ability to produce CQDs in large quantities with relatively high yields. For instance, studies have shown that pyrolysis of citric acid at 300 °C for 2 hours can produce CQDs with a QY of up to 80%, depending on the surface passivation and functionalization. Similarly, biomass-derived CQDs synthesized *via* pyrolysis have demonstrated QYs ranging from 10% to 50%, depending on the source material and pyrolysis conditions. The process is also versatile, as it can be adapted to various carbon precursors, including waste materials like fruit peels, coffee grounds, and agricultural residues, making it an eco-friendly and cost-effective approach. For example, pyrolysis of banana peel at 400 °C for 4 hours has been reported to produce CQDs with a QY of 18%, suitable for applications in bioimaging and sensing. Despite its advantages, pyrolysis has some limitations. The high temperatures required can lead to the formation of by-products or impurities, necessitating post-synthesis purification steps such as dialysis or centrifugation. Additionally, the process may result in CQDs with broad size distributions, which can affect their optical properties. However, these challenges can be mitigated by optimizing the pyrolysis conditions and using surface passivation agents (*e.g.*, polyethylene glycol or amines) to enhance the fluorescence and stability of the CQDs. Overall, pyrolysis remains a robust and scalable method for synthesizing CQDs, with applications ranging from bioimaging and sensing to energy storage and catalysis.^38^

#### 3.2.5. Thermal decomposition

Thermal decomposition is a widely used method for synthesizing CQDs, which are nanoscale carbon-based materials with unique optical and electronic properties. This process involves the controlled heating of carbon-rich precursors, such as citric acid, glucose, or other organic compounds, at high temperatures (typically between 150 °C and 300 °C) in an inert atmosphere or under vacuum. During heating, the precursor molecules undergo carbonization, breaking down into smaller carbon fragments that eventually nucleate and grow into CQDs. The size, structure, and surface properties of the resulting CQDs can be tuned by adjusting parameters such as temperature, heating duration, and the choice of precursor. For example, citric acid is a commonly used precursor, and heating it at around 200 °C for 30–60 minutes typically yields CQDs with sizes ranging from 2 to 10 nm and quantum yields of up to 80%.^39^

One of the key advantages of thermal decomposition is its simplicity and scalability, as it does not require complex equipment or harsh chemicals. The method also allows for the incorporation of heteroatoms into the CQD structure by using precursors containing these elements, which can enhance the optical and catalytic properties of the CQDs. For instance, nitrogen-doped CQDs synthesized *via* thermal decomposition have shown improved photoluminescence and electrochemical performance compared to undoped CQDs. Additionally, the surface of CQDs can be functionalized during or after the synthesis process to improve their solubility, stability, and compatibility with specific applications. Studies have demonstrated that CQDs produced *via* thermal decomposition exhibit excellent biocompatibility, making them suitable for biomedical applications such as bioimaging and drug delivery.^40^

#### 3.2.6. Carbonization of biomass

Carbonization of biomass is an eco-friendly and cost-effective method for synthesizing CQDs by converting organic biomass into carbon nanomaterials. This process involves the pyrolysis of carbon-rich biomass, such as plant waste, fruit peels, or agricultural residues, at high temperatures in the absence of oxygen. The carbonization breaks down the complex organic structures, forming CQDs through carbonization and surface functionalization. Typical carbonization conditions include heating the biomass at 180 °C to 400 °C for several hours, which results in small, fluorescent carbon nanoparticles. These CQDs can exhibit various optical properties, such as strong fluorescence, depending on the type of biomass used and the pyrolysis conditions. For example, CQDs derived from orange peel carbonized at 300 °C for 4 hours have been reported to have a particle size of 3.2 nm and blue fluorescence under UV light.^41^ One of the key benefits of biomass-derived CQDs is their versatility and sustainability. Biomass materials are abundant and renewable, making them an attractive alternative to synthetic precursors. Moreover, the surface of CQDs can retain functional groups, such as hydroxyl and carboxyl groups, which enhance their solubility and biocompatibility. This makes CQDs suitable for a wide range of applications. This method not only addresses the growing need for sustainable material production but also yields highly functional nanomaterials with promising properties for optical, energy, and biomedical applications.

### 3.3. Green synthesis

#### 3.3.1. Biomass-derived CQDs

Carbonization of biomass to produce CQDs involves converting organic materials, such as fruit peels or plant waste, into carbon-based nanomaterials through pyrolysis, where the biomass is heated in the absence of oxygen. This process breaks down complex molecules into smaller carbon structures, forming CQDs, typically less than 10 nm in size with unique properties like fluorescence. The temperature during pyrolysis, usually between 180 °C and 400 °C, significantly influences the characteristics of the CQDs. For example, carbonizing citric acid can yield CQDs with a quantum yield of up to 13.2%, while biomass like orange peel, carbonized at 300 °C for 4 hours, produces CQDs with a diameter of 3.2 nm and blue fluorescence under UV light. These CQDs are also enhanced with functional groups such as hydroxyl and carboxyl, which improve their solubility and biocompatibility, making them ideal for applications in bioimaging, drug delivery, and sensors. Additionally, using natural and recyclable biomass resources helps reduce waste and pollution, contributing to environmental sustainability.^42^

However, challenges exist in controlling the properties of the CQDs, as factors like biomass type, carbonization temperature, and time can significantly affect the final product. This can result in variations in the size, fluorescence, and overall quality of the CQDs, sometimes not meeting expectations. Moreover, the efficiency and yield of the process can be inconsistent, requiring further optimization to improve the quality and uniformity of the CQDs. Despite these drawbacks, biomass carbonization remains a promising and cost-effective method for producing CQDs, with potential applications in energy, environmental, and medical fields, offering an eco-friendly alternative to conventional nanomaterial production methods.^43^

#### 3.3.2. Enzymatic synthesis

Enzymatic synthesis of CQDs involves using enzymes to catalyze the conversion of organic precursors, such as sugars, proteins, or amino acids, into carbon-based nanomaterials. This method operates under mild conditions, typically at temperatures between 30 °C and 80 °C, and uses enzymes like horseradish peroxidase or laccase to facilitate the breakdown of organic molecules. For example, enzymatic synthesis of CQDs from glucose using horseradish peroxidase has been shown to produce CQDs with a quantum yield of approximately 8.5%, with the size of the CQDs ranging from 2 to 6 nm. These CQDs exhibit excellent fluorescence properties, making them ideal for applications in bioimaging, drug delivery, and biosensors. The surface of the CQDs is often functionalized with hydroxyl, carboxyl, and amino groups, enhancing their water solubility and biocompatibility. The primary advantages of enzymatic synthesis are the environmentally friendly and energy-efficient conditions under which it is carried out. Since the process occurs at lower temperatures (usually below 100 °C) and in aqueous environments, it reduces the need for toxic chemicals or high-energy inputs. Additionally, enzymatic methods can lead to CQDs with highly controlled surface properties and improved biocompatibility. For example, CQDs synthesized from amino acids using laccase enzymes have shown excellent biocompatibility and stability, with quantum yields of up to 10%. However, challenges remain in the scalability and cost of enzyme production, and enzyme deactivation can occur under prolonged exposure to harsh conditions.^44^

### 3.4. Chemical vapor deposition (CVD)

CVD is a widely used method for synthesizing CQDs through the deposition of carbon-containing gases onto a heated substrate in a reaction chamber. The process begins by introducing gaseous precursors, such as methane, acetylene, or ethylene, into a chamber that is heated to temperatures typically between 500 °C and 1000 °C. Under these conditions, the carbon-containing gases decompose, and carbon atoms are deposited onto the surface of the substrate, forming carbon-based nanomaterials, including CQDs. The size, morphology, and optical properties of the CQDs can be controlled by adjusting key parameters such as temperature, precursor flow rate, pressure, and the type of catalyst used. For example, CQDs produced from methane using CVD can range in size from 2 to 10 nm and exhibit quantum yields up to 15%. This method allows for precise tuning of CQD properties by controlling the deposition process.^45^

One of the main advantages of CVD is its ability to produce high-quality CQDs with excellent structural uniformity, high crystallinity, and strong optical properties. The method is versatile and can accommodate various precursors, enabling the production of CQDs with specific surface functionalities by incorporating different functional gases during the deposition process. This makes CVD a promising approach for applications in bioimaging, sensors, and energy storage. Additionally, CVD can be scaled up for industrial production, making it suitable for mass production of CQDs with consistent quality. CQDs produced *via* CVD typically exhibit high photostability and fluorescence, which are critical for various technological applications. For instance, CQDs synthesized from acetylene through CVD have shown strong blue fluorescence and exceptional stability.^46^ Although CVD offers many advantages, it also presents some challenges. One of the primary concerns is the high energy consumption due to the need for elevated temperatures and specialized equipment, which can increase production costs. Additionally, while the CVD process allows for precise control over CQD properties, achieving consistent results can still be challenging due to slight variations in the reaction conditions. However, these challenges are often outweighed by the high-quality CQDs produced and the ability to tailor their properties for specific applications. With proper optimization, CVD remains an effective and scalable method for producing high-performance CQDs for use in advanced fields such as electronics, biosensing, and energy applications.^45,46^

### 3.5. Plasma treatment

Plasma treatment is a surface modification technique used to functionalize CQDs by exposing them to a low-temperature plasma environment. In this process, gaseous precursors like oxygen, nitrogen, or argon are ionized to form plasma, which produces highly reactive species such as ions, radicals, and excited atoms. When CQDs are exposed to this plasma, the reactive species interact with the surface of the CQDs, introducing new functional groups such as hydroxyl, carboxyl, or amino groups. The treatment typically occurs under mild conditions, with plasma power ranging from 10 to 100 W and treatment times between 1 and 30 minutes, depending on the specific application. For example, oxygen plasma treatment can introduce carboxyl groups onto the surface of CQDs, improving their water solubility and dispersibility. In one study, oxygen plasma treatment of CQDs led to a significant increase in their surface carboxyl content, enhancing their biocompatibility and making them more suitable for biomedical applications such as drug delivery and bioimaging.^47^

The process of plasma treatment allows for precise control over the surface chemistry of CQDs without affecting their bulk structure. The type of gas used in the plasma, the plasma power, and the treatment time all influence the type and density of the functional groups introduced. Oxygen plasma, for instance, is effective at introducing oxygen-containing groups such as carboxyl and hydroxyl, which improve the hydrophilicity and biocompatibility of the CQDs. Nitrogen plasma, on the other hand, can introduce amino groups, which further enhance the compatibility of CQDs with biological systems. By adjusting the plasma parameters, the optical properties of CQDs, such as fluorescence intensity and emission wavelength, can also be tuned. For example, plasma treatment has been shown to improve the fluorescence quantum yield of CQDs by up to 20%, making them more suitable for use in sensors and bioimaging applications.^48^ While plasma treatment offers significant advantages, there are challenges in its application. One of the key challenges is the potential for over-oxidation of the CQDs during prolonged exposure to oxygen plasma, which could degrade their fluorescence properties. For instance, excessive oxidation can lead to the formation of defects in the CQD structure, which may reduce their photostability and fluorescence. Therefore, precise control over the plasma exposure time and power is essential to achieve the desired functionalization without compromising the performance of CQDs. The scalability of the plasma treatment process is another challenge, as it often requires specialized equipment, such as a plasma reactor, which may not be cost-effective for large-scale production. However, plasma treatment remains an attractive approach for fine-tuning the surface properties and enhancing the functionality of CQDs for a wide range of applications, including environmental sensing, drug delivery, and biosensing.^47,48^

### 3.6. Scalability considerations for CQD synthesis

The synthesis of CQDs can be broadly classified into top-down and bottom-up approaches, each with distinct implications for scalability. Top-down methods, such as laser ablation, arc discharge, and electrochemical oxidation, excel in producing high-purity CQDs with well-defined properties. However, their scalability is limited by high energy consumption, the need for specialized equipment, and relatively low yields. For instance, laser ablation typically yields 5–15 mg of CQDs per gram of precursor, while arc discharge generates mixed carbon byproducts requiring extensive purification, both of which hinder large-scale production. Conversely, bottom-up methods, including hydrothermal, solvothermal, and microwave-assisted synthesis, offer greater potential for scale-up. These techniques utilize simple setups and cost-effective and abundant precursors (*e.g.*, citric acid, glucose, or biomass), and can achieve higher yields under mild conditions. Hydrothermal synthesis, for example, has been widely adopted due to its eco-friendliness and ability to produce CQDs in bulk, with quantum yields up to 80% when optimized. Microwave-assisted synthesis further enhances scalability by reducing reaction times to minutes, enabling rapid production with consistent quality. Green synthesis routes, such as carbonization of biomass, also align with industrial needs by leveraging renewable resources and minimizing waste. While bottom-up methods may require post-processing to ensure uniformity, their simplicity, lower cost, and adaptability make them more suitable for large-scale CQD production, particularly for electrocatalytic applications in sustainable energy technologies. To provide a clearer comparison, Table 1 summarizes the scalability of representative top-down and bottom-up methods based on key metrics such as cost, yield, and equipment requirements, supported by literature references.

**Table 1:** Comparison of scalability for top-down and bottom-up CQD synthesis methods

Synthesis method	Approach	Cost	Yield	Equipment complexity	Scalability potential	Key limitations	References
Laser ablation	Top-down	High	Low (5–15 mg g^−1^)	High (pulsed lasers)	Low	High energy use and low yield	21 and 22
Arc discharge	Top-down	High	Moderate	High (arc setup)	Low	Byproduct separation and energy cost	23 and 24
Electrochemical oxidation	Top-down	Moderate	Moderate (variable)	Moderate (cell setup)	Moderate	Purification needs and a slower process	25 and 26
Hydrothermal synthesis	Bottom-up	Low	High (up to 80% QY)	Low (autoclave)	High	Uniformity requires optimization	31 and 32
Microwave-assisted synthesis	Bottom-up	Low	High	Low (microwave)	High	Possible aggregation if uncontrolled	35 and 36
Carbonization of biomass	Bottom-up	Very low	Moderate to high	Low (furnace)	High	Variability in precursor quality	41 and 42

## 4. Applications in electrocatalysis

### 4.1. Oxygen evolution reaction

The oxygen evolution reaction (OER) is a critical electrochemical process in energy conversion systems, such as water splitting for hydrogen production. It involves the oxidation of water molecules to form oxygen gas, protons, and electrons. In the context of CQDs, the OER occurs at the surface of these nanomaterials, where the CQDs act as electrocatalysts. The mechanism typically proceeds through several steps: adsorption of water molecules, oxidation of water to produce hydroxyl radicals (OH˙), followed by the formation of oxygen intermediates, and finally the release of molecular oxygen. CQDs, due to their high surface area, tunable electronic properties, and rich surface functionalization, enhance the reaction kinetics by lowering the activation energy and improving charge transfer, thus increasing the efficiency of the OER.^49^

The surface chemistry of CQDs plays a crucial role in their catalytic activity for the OER. Functional groups such as carboxyl, hydroxyl, and carbonyl groups on the surface of CQDs can act as active sites, enhancing the adsorption of water molecules and the formation of reaction intermediates. This interaction improves the efficiency of the electron transfer required for the OER. The improved performance of CQDs decorated on Ba_0.5_Sr_0.5_Co_0.8_Fe_0.2_O_3−*δ*_ perovskite nanofibers was demonstrated, with the CQDs@BSCF-NFs catalyst achieving a low overpotential of 0.35 V at 10 mA cm^−2^, outperforming both individual BSCF-NFs and commercial IrO_2_. The catalyst demonstrated an outstanding current density of 140.8 mA cm^−2^ at a potential of 1.65 V *vs.* RHE, approximately 5 and 19 times higher than that of BSCF-NFs and IrO_2_, respectively. This significantly enhanced activity was attributed to the enlarged specific surface area, increased surface oxygen vacancies, and the synergy between CQDs and BSCF. Moreover, the CQDs@BSCF-NFs catalyst exhibited high electrocatalytic stability for 10 h of operation.^50^

**Fig. 3:**
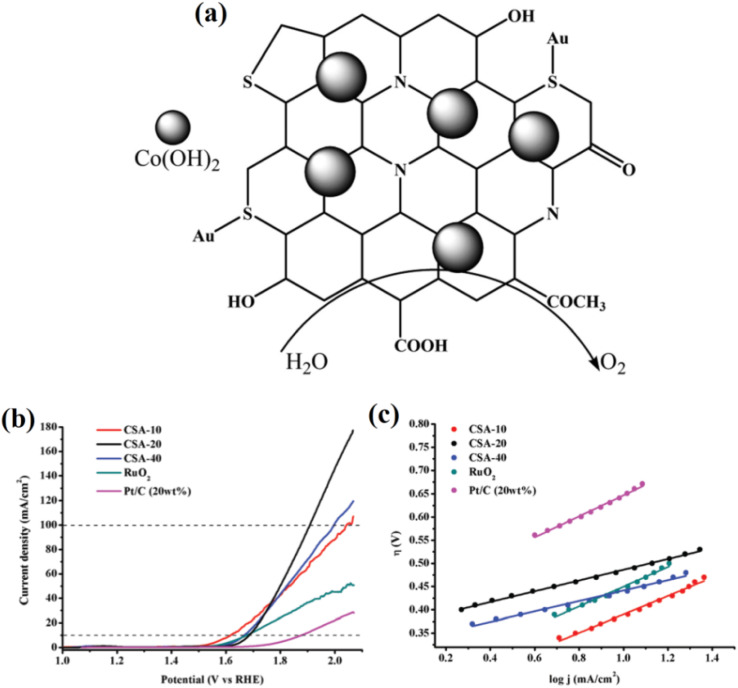
(a) Schematic diagram of OER activity enhancement by Co(OH)_2_ incorporated Au-doped carbon quantum dots (Au-SCQDs), (b) LSV curve for OER studies, and (c) Tafel plots of Co(OH)_2_-Au-SCQDs. Reproduced from ref. 51 with permission from the Royal Society of Chemistry.

Beyond standalone CQDs, their combination with other catalytic materials has led to substantial improvements in the OER. For instance, CQDs doped with metals such as gold have exhibited enhanced fluorescence and OER catalytic activity when combined with cobalt hydroxide. This hybrid approach alters the electronic properties of CQDs, enabling them to facilitate more efficient charge transfer during the OER. The gold doping approach helps reduce the overpotential and increases the overall catalytic performance of the system. Furthermore, the incorporation of heteroatoms such as nitrogen into CQDs has been shown to further improve their catalytic performance. Nitrogen doping increases the electron density on the surface of CQDs, which helps stabilize intermediates and improve charge transfer during the OER. This is consistent with studies by Muthukumar and Anthony, where the presence of nitrogen in CQDs enhanced their catalytic properties when coupled with transition metal oxides such as cobalt hydroxide.^51^ In Fig. 3a, the schematic representation depicts the structural integration of Co(OH)_2_ with S,N-co-doped CQDs anchored on a conductive carbon substrate. The CQDs are functionalized with various oxygenated groups (*e.g.*, COOH and OH), which improve hydrophilicity and charge transport. Doping with sulfur and nitrogen introduces additional active sites and modifies the electronic structure of the CQDs, enhancing their affinity for oxygenated intermediates. The uniform distribution of Co(OH)_2_ nanoparticles over the CQDs boosts the intrinsic catalytic activity, as the synergistic interaction between the Co(OH)_2_ and the CQDs facilitates more efficient adsorption and the conversion of water molecules during the OER. Fig. 3b displays the LSV curves for various CSA-*x* samples (where CSA refers to Co(OH)_2_–S,N-CQD–Au hybrids, and *x* denotes the volume of the CQD precursor), RuO_2_, and commercial Pt/C in 1.0 M KOH. CSA-30 shows superior OER performance with a much lower overpotential to reach 10 mA cm^−2^ compared to the others, indicating optimized Co(OH)_2_ loading and a balanced interaction with the CQDs. Fig. 3c presents the corresponding Tafel plots, further confirming the enhanced kinetics of CSA-30 with the lowest Tafel slope, indicative of faster charge transfer and improved reaction kinetics. The collective data emphasize how S,N-doped CQDs synergistically enhance the catalytic activity of Co(OH)_2_, validating their promising role in OER electrocatalysis.

Nitrogen-doped CQDs, when integrated with NiFe oxide clusters, demonstrated exceptional performance in the OER, with a significantly lower overpotential compared to non-doped CQDs. Specifically, the NiFeO_*x*_ clusters strongly coupled with the N-doped carbon (NiFeO_*x*_©NC) hybrid, prepared by a one-step electrodeposition process, achieved a low overpotential of 195 mV at 10 mA cm^−2^, outperforming the commercial RuIr-based dimensionally stable anode. In addition to this, the NiFeO_*x*_©NC hybrid exhibited faster kinetics, with a Tafel slope of 33 mV dec^−1^, and demonstrated excellent long-term durability, rate capability, and environmental adaptability, highlighting the potential of this approach for advancing clean electrochemical technology.^52^

This method involves the electrostatic interaction of Ni^2+^ and Fe^3+^ ions with nitrogen-doped CQDs (N-CQDs), resulting in the formation of NiFe clusters on the carbon support. This hybrid material serves as the OER-active anode in water splitting. The integration of NiFe clusters with N-CQDs enhances the material's electrochemical properties, creating a highly efficient catalyst for the OER. The uniform distribution of NiFeO_*x*_ nanoparticles is beneficial for maximizing the contact area between the catalyst and the electrolyte, which leads to better catalytic activity for water splitting. The nanoparticles, with a relatively uniform size range of 10–20 nm as shown in the inset, are well-dispersed across the N-CQD matrix. This nanoscale size and even dispersion are critical for providing more active sites for the OER, contributing to improved catalytic efficiency. The NiFeO_*x*_©NC catalyst shows significantly higher current density at lower overpotentials compared to the other catalysts, demonstrating its superior activity for the OER. The enhanced performance can be attributed to the synergistic effect between the NiFe clusters and the N-CQD support, which optimizes the electronic structure and increases the availability of active sites for the reaction.

The stability of CQDs during the OER is another important factor for their practical application. Research has shown that CQDs can maintain their structural integrity and catalytic performance over extended periods, making them suitable for real-world energy conversion applications. For example, platinum nanoparticles supported on CQDs have been found to show sustained OER activity, indicating that CQDs contribute not only to catalytic efficiency but also to long-term stability. The as-prepared Pt/CQD nanocomposites demonstrated excellent electrochemical performance, with a stable ORR activity after 1000 cycles, and improved charge transfer characteristics. The strong interaction between the nanosized platinum particles and CQDs was shown to modulate the electronic structure of the platinum clusters, enhancing both catalytic efficiency and stability.^53^ In addition to their catalytic stability, CQDs offer significant cost advantages. Traditional OER catalysts such as platinum and iridium are expensive and scarce, limiting their large-scale use. CQDs, derived from carbon sources, are far more abundant and can be synthesized through low-cost, environmentally friendly methods. This makes CQDs a highly attractive material for large-scale renewable energy applications. Research demonstrated that CQDs derived from coal, a carbon-rich source, could deliver excellent catalytic activity for the OER, further reinforcing the economic feasibility of using CQDs in energy conversion systems.

Specifically, a bottom-up strategy was developed using coal-based CQDs to synthesize uniform metal alloy nanoparticles embedded in a carbon matrix. The resulting electrocatalyst showed impressive OER performance, highlighting the potential of coal as a low-cost, sustainable precursor for high-quality nano-catalysts.^54^ CQDs have also been explored for their potential in multifunctional catalytic applications. For example, the combination of CQDs with zinc cobalt oxide (ZnCo_2_O_4_) not only enhanced the OER performance but also enabled the catalyst to degrade organic pollutants in wastewater. This bifunctional catalyst highlights the dual role of CQDs in energy production and environmental remediation, creating a dual-purpose material that improves sustainability in both fields. Specifically, the 0.7 CQDs/ZnCo_2_O_4_ composite exhibited superior photocatalytic performance, achieving over 100% decomposition of methylene blue (MB) and 96% decomposition of methylene orange (MO) dyes under UV light after 50 and 140 minutes, respectively. The enhanced performance was attributed to the improved charge transfer rate and synergistic effect between the CQDs and ZnCo_2_O_4_. Additionally, this composite displayed a very low overpotential of 220 mV at 100 mA cm^−2^ for OER activity, with excellent durability for 250 hours at 500 mA cm^−2^, demonstrating its potential in both energy and environmental applications.^55^

Moreover, CQDs have been integrated into photoelectrochemical systems for solar-driven water splitting. For instance, CQDs coupled with TiO_2_ as a photoanode have been shown to significantly improve charge separation and reduce recombination, leading to higher photocatalytic efficiency for water splitting under visible light. Such photoelectrochemical systems offer promising solutions for solar-driven hydrogen production, contributing to the overall advancement of renewable energy technologies. In another study, CQDs were coupled with Fe_2_O_3_ nanowires, resulting in a 27-fold increase in photocurrent density at 0.23 V *vs.* Ag/AgCl, compared to pristine Fe_2_O_3_ nanowires. The enhanced photoelectrochemical performance was attributed to the improved optical absorption, accelerated interfacial charge carrier transfer, and effective separation of photogenerated electron–hole pairs induced by the CQD decoration, demonstrating the potential of CQDs in advancing solar-driven water splitting for clean energy production.^56^

A more recent innovation involves using CQDs as scaffolds for metal oxide growth. For instance, α-Fe_2_O_3_ (hematite) growth on CQD scaffolds has led to the creation of photoanodes with improved photocatalytic activity for water splitting under visible light. The CQDs not only serve as a template for metal oxide growth but also facilitate charge transfer, thus enhancing the overall photoelectrochemical performance. In a recent study, CQDs were used as conductive nano-scaffolds for the growth of α-Fe_2_O_3_ nanoparticulates on a Ti substrate, significantly enhancing the photocurrent response. The resulting Ti/CQDs@α-Fe_2_O_3_ photoanode exhibited a photocurrent density of 2.1 mA cm^−2^ at an applied bias of +0.5 V *vs.* Ag/AgCl, which was 10 times higher than that of the Ti/α-Fe_2_O_3_ photoanode. This improvement was attributed to enhanced charge-transfer rates and the suppression of electron–hole recombination due to the increased hole-diffusion length in the conducting nano-scaffold structure. Furthermore, the Ti/CQDs@α-Fe_2_O_3_ photoanode demonstrated high stability, with minimal change (±5%) in photocurrent after 4 hours of visible light irradiation, showcasing the durability and effectiveness of the CQDs as scaffolds for metal oxide growth.^57^

**Fig. 4:**
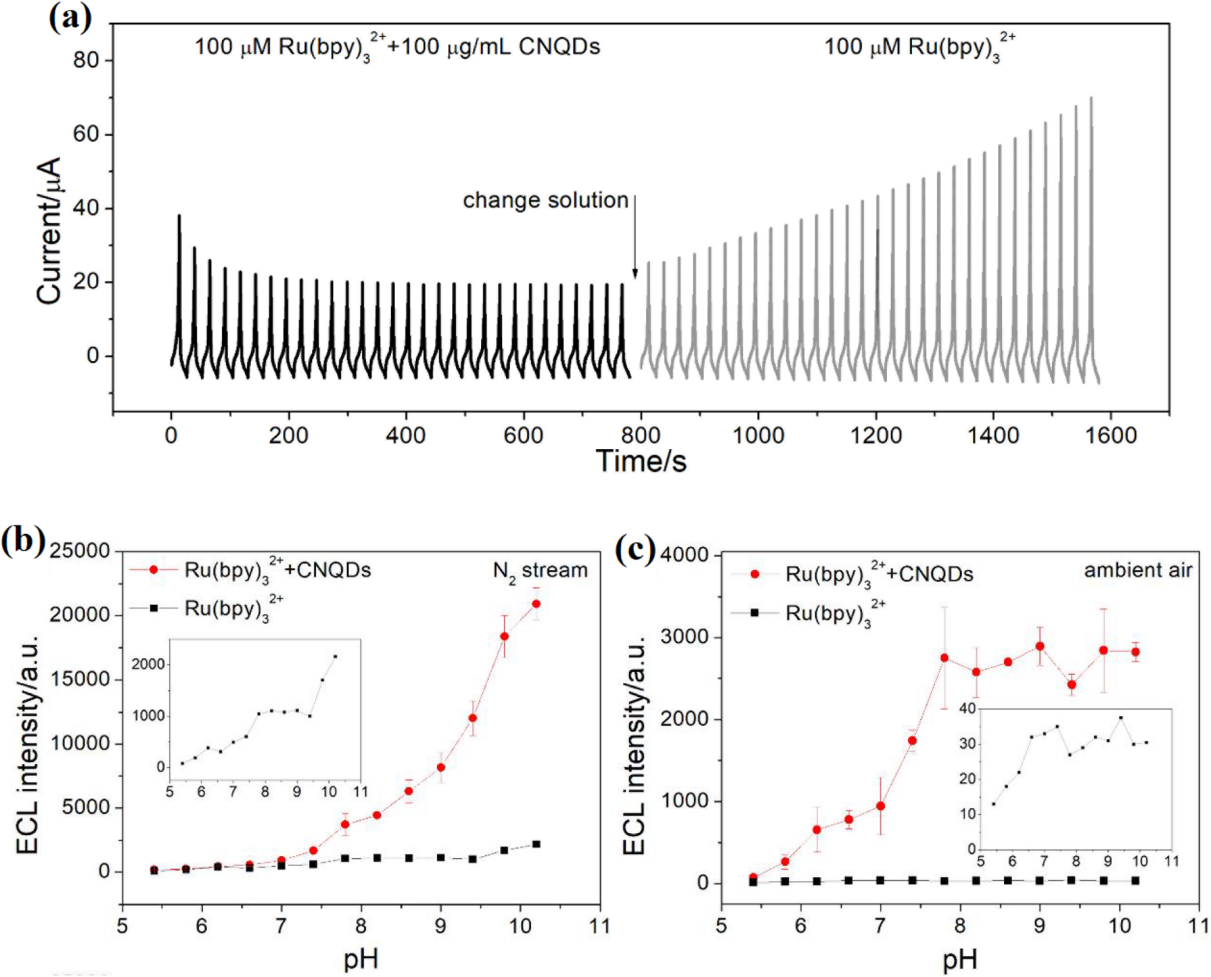
(a) Chronoamperometric ECL response of Ru(bpy)_3_^2+^ with and without CNQDs during solution exchange. pH-dependent ECL intensity of Ru(bpy)_3_^2+^ with and without CNQDs under N_2_ (b) and ambient air (c) atmospheres, highlighting enhanced emission with CNQDs, especially at alkaline pH. Reproduced with permission from ref. 59, copyright 2020 American Chemical Society.

In addition to their use in energy applications, CQDs can also be engineered to modulate electrocatalytic reactions. For example, CQDs functionalized with phosphorene can act as efficient electrocatalysts for the OER, offering a novel way to enhance catalytic activity by combining quantum dots with layered materials. Additionally, in a recent study, nitrogen-doped CQDs were used in composite aerogels for energy storage systems, demonstrating enhanced electrochemical properties. The N-CQDs/reduced GO/porous Fe_2_O_3_ (N-CQDs/rGO/Fe_2_O_3_) composite aerogels exhibited excellent rate capability and superior cycling performance with 80.4% capacity retention after 5000 cycles at 3 A g^−1^. The synergy between N-CQDs, rGO, and Fe_2_O_3_ contributed to the impressive electrochemical properties, showcasing the potential of CQD-based composites for enhancing electrocatalytic reactions.^58^ In another study, carbon nitride quantum dots (CNQDs) were found to inhibit the OER in certain electrocatalytic environments. The interaction between CNQDs and ruthenium-based catalysts was shown to enhance electrochemiluminescence (ECL) in anodic systems. This approach alleviated energy losses due to nonradiation relaxation in the OER process, leading to a significant enhancement in anodic ECL. In the Ru(bpy)_3_^2+^/CNQD system, CNQDs enhanced the anodic ECL of Ru(bpy)_3_^2+^ 10-fold in a nitrogen stream and 161-fold in ambient air. These findings underscore the potential of CNQDs in optimizing various aspects of catalytic performance, including selectivity and efficiency in energy conversion reactions. The system also showed advantages compared to traditional coreactant ECL systems, such as requiring a low dosage of CNQDs (100 μg mL^−1^) and exhibiting favorable regeneration capacity. This strategy provides a new pathway to enhance electrocatalytic performance and improve the efficiency of energy conversion reactions.^59^Fig. 4a presents the current–time profile of ECL under repeated potential cycling for Ru(bpy)_3_^2+^ with and without carbon nitride quantum dots (CNQDs). When 100 μM Ru(bpy)_3_^2+^ is combined with 100 μg per mL CNQDs (black trace), a sequence of increasing ECL peaks is observed, followed by gradual signal attenuation over time. Upon switching to a solution containing only Ru(bpy)_3_^2+^ (gray trace), the ECL response diminishes significantly and remains relatively stable. This behavior highlights the crucial role of CNQDs in enhancing ECL intensity through surface-mediated redox processes. These enhancements are attributed to the generation of ROS or oxidized intermediates at the CNQD surface, closely resembling mechanisms seen in the OER. Fig. 4b and c further show the ECL intensity across different pH values under nitrogen-purged (b) and ambient air (c) conditions. In both environments, the Ru(bpy)_3_^2+^/CNQDs system shows superior ECL intensity compared to Ru(bpy)_3_^2+^ alone, particularly at higher pH (8–11). The enhancement is more significant under N_2_, suggesting that the presence of O_2_ in air modulates or competes with electron-transfer processes. The strong pH dependence and response under oxygen-controlled conditions indicate that CNQDs not only serve as efficient ECL co-reactants but also exhibit electrocatalytic properties reminiscent of the OER, especially in alkaline media where the OER is thermodynamically favorable.

The use of CQDs in combination with layered double hydroxides (LDHs), such as NiCo-LDH, has been explored for both OER and supercapacitor applications. The hybrid system of CQDs and NiCo-LDH demonstrated a significant enhancement in both energy storage and OER efficiency. The CQDs not only improved the electronic conductivity of the LDH but also increased the overall surface area, providing more active sites for catalytic reactions. In one study, the CQD-decorated NiCo-LDH electrodes generated a current density of 10 mA cm^−2^ at an overpotential of 226 mV, compared to 332 mV for bare NiCo-LDH. The CQD/NiCo-LDH system also exhibited remarkable specific capacitance values of 1136 F g^−1^ at 1 A g^−1^ current density and 441 F g^−1^ at 10 A g^−1^, with nearly 62% capacitance retention after 500 charge–discharge cycles.^60^ This demonstrates the potential of CQDs to bridge energy technologies, enhancing both OER and supercapacitor performance.

Green-synthesized CQDs have emerged as a promising material for enhancing photocatalytic reactions, particularly the OER. Recent research demonstrated that CQDs, synthesized *via* microwave irradiation with glycerol as a solvent and copper ions as a cation promoter, significantly improve the OER performance. When integrated with TiO_2_ nanoparticles, these CQDs exhibited over 12 times higher OER efficiency compared to the HER. The enhanced photocatalytic activity is attributed to the high electronic conductivity of the Cu-doped CQDs, which facilitate efficient electron transfer during the OER process. Additionally, the Cu-CQDs@TiO_2_ composite demonstrated visible light activity, achieving complete degradation of the indigo carmine dye in less than three hours, showing its potential for environmental applications like water purification. These findings underline the value of green-synthesized CQDs in advancing clean energy solutions, particularly in water splitting and solar-driven photocatalysis, while also providing a sustainable alternative for energy production and environmental remediation.^61^ Recent advancements in CQD-based electrocatalysts have focused on leveraging the synergy between CQDs and metal ions or metal oxides to enhance electrocatalytic performance. In one notable study, CQDs were co-doped with Co and Fe and employed as OER electrocatalysts, exhibiting impressive activity with a low overpotential of 320 mV. Moreover, when combined with Ru in a CoFeRu@C composite, the catalyst demonstrated an extremely low overpotential of just 4 mV for the HER. This dual-functional electrocatalyst approach highlights the potential of CQDs and multi-metal doping in developing high-performance catalysts for energy conversion processes. Furthermore, the study presented an innovative one-pot hydrothermal method to synthesize these catalysts, offering a scalable and cost-effective route for large-scale industrial applications.^62^

### 4.2. Hydrogen evolution reaction

The hydrogen evolution reaction (HER) plays a crucial role in hydrogen production, which is a cornerstone of clean energy technologies like water electrolysis and fuel cells. This reaction involves the reduction of protons to form hydrogen gas at the cathode in acidic or neutral electrolytes. Efficient catalysts are necessary to lower the activation energy of the reaction and increase the rate of hydrogen production. CQDs have emerged as promising candidates for the HER due to their unique electronic properties, high surface area, and tunable surface functionalities, which provide abundant active sites for proton adsorption and electron transfer.^63,64^ Surface modifications, including the incorporation of functional groups like –OH, –COOH, and –NH_2_ groups, further enhance the catalytic properties of CQDs by improving proton adsorption and facilitating electron transfer. For instance, Chattopadhyay and Bag (2024) studied how surface functionalization of CQDs enhances their electrocatalytic performance. They found that functionalizing the surface of CQDs improves proton adsorption, a crucial step in the HER, where protons from the electrolyte are adsorbed onto the catalyst surface before being reduced to H_2_. This enhancement in proton adsorption directly correlates with better catalytic efficiency, as it facilitates faster and more effective hydrogen generation.^65^ These results underscore the importance of surface engineering in electrochemistry to optimize the interaction between catalysts and reactants. Additionally, research has highlighted the kinetic parameters of CQDs for the HER, emphasizing that surface functionalization plays a critical role in enhancing catalytic performance. Their study demonstrated that functionalized CQDs exhibit improved charge transfer properties, directly contributing to more efficient HER. Moreover, they found that surface functionalization lowers overpotentials, a key factor in improving the efficiency of HER catalysts. This research provides valuable insight into how optimizing the surface characteristics of CQDs can enhance their catalytic activity, particularly in water splitting for clean hydrogen production. Furthermore, it underscores the potential of CQDs as highly efficient electrocatalysts for future energy applications, especially in the context of sustainable hydrogen production.^63^

The incorporation of heteroatoms such as nitrogen into CQDs has been shown to enhance their HER performance by improving electron density and facilitating proton interaction. Research has demonstrated that nitrogen-doped CQDs significantly improve HER activity by introducing additional electronic states that facilitate proton adsorption and electron transfer, thus accelerating the reduction process. Moreover, studies have explored the synergy between metal catalysts and carbon-based supports, showing that Ru nanoparticles (Ru-NPs) supported on CQDs exhibit superior HER performance compared to other carbon supports. The enhanced performance is attributed to strong coordination interactions between the d orbitals of Ru and the surface functional groups of CQDs, which not only confine the Ru-NPs but also prevent their aggregation. These findings provide valuable insight into designing cost-effective and high-performance Ru-based electrocatalysts for the HER, highlighting the importance of both heteroatom doping and the interaction between metal and carbon supports in boosting catalytic activity.^64^

**Fig. 5:**
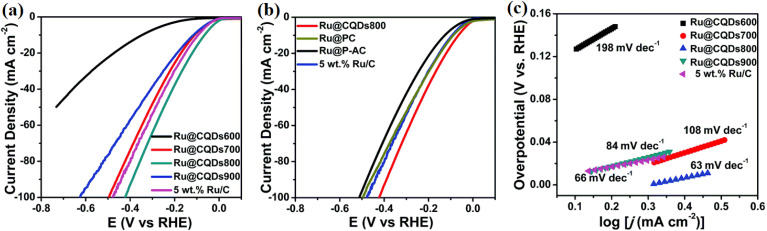
(a) HER polarization curves for the Ru@CQDs annealed at different temperatures; (b) HER polarization curves with different carriers in 1 M KOH; Tafel plots (c) for the Ru@CQDs annealed at different temperatures. Reproduced from ref. 64 with permission from the Royal Society of Chemistry.

Fig. 5 shows the electrocatalytic performance of Ru nanoparticles embedded in carbon quantum dots (Ru@CQDs) for the HER in an alkaline medium (1 M KOH), highlighting the impact of annealing temperature and carbon supports. In panel (a), HER polarization curves reveal that the catalytic activity is strongly dependent on the annealing temperature. Among the tested samples (500–900 °C), Ru@CQDs800 exhibits the most negative onset potential and the highest current density, indicating superior HER activity. This enhanced performance is due to the optimal balance between particle size, conductivity, and defect density at 800 °C, which improves electron transfer and active site accessibility. Panel (b) compares HER activity for Ru@CQDs800 supported on various carbon carriers, including PC (porous carbon), AC (activated carbon), and commercial 5 wt% Ru/C. The Ru@CQDs800 sample again shows the best performance, suggesting that the intrinsic structure of the CQDs offers a more favorable environment for the HER than conventional carbon supports. The homogeneous distribution of Ru nanoparticles and the conductive, defect-rich CQD matrix contribute to this improved catalytic behavior by facilitating charge transport and enhancing the hydrogen adsorption kinetics. Panel (c) presents the corresponding Tafel plots, which provide insights into the HER kinetics. The Ru@CQDs800 sample achieves the lowest Tafel slope (63 mV dec^−1^), indicating faster HER kinetics and more favorable reaction pathways. In contrast, Ru@CQDs500 and Ru@CQDs700 show higher Tafel slopes (198 and 108 mV dec^−1^, respectively), reflecting sluggish kinetics. These findings confirm that annealing at 800 °C produces an optimal nanostructure for efficient hydrogen evolution, underscoring the role of CQDs not only as structural supports but also as active components that contribute to the catalytic mechanism.

**Fig. 6:**
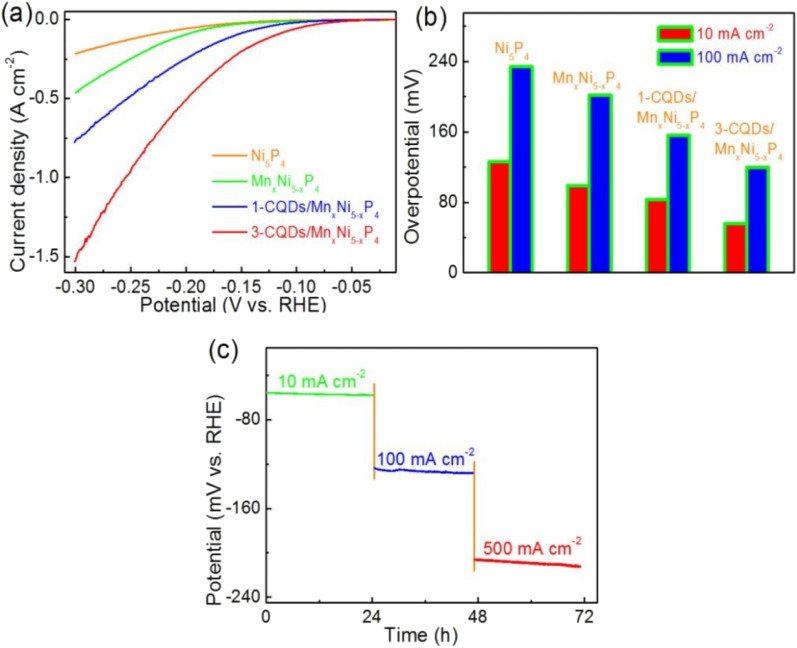
Electrocatalytic performance of different catalysts in 1 M KOH. (a) Polarization curves, (b) overpotentials at current densities of 10 and 100 mA cm^−2^, and (c) chronopotentiometry curves of 3-CQDs/Mn_*x*_Ni_5−*x*_P_4_ at different current densities for the HER. Reproduced from ref. 66 with permission from the Royal Society of Chemistry.

Combining CQDs with other materials, particularly transition metal phosphides, has proven to be an effective strategy for enhancing HER performance. For example, the addition of CQDs to manganese–nickel phosphide significantly improves HER activity by providing additional active sites and facilitating better charge transfer. In one study, the CQD-functionalized manganese–nickel phosphide catalyst demonstrated a current density of 30 mA cm^−2^ at an overpotential of 150 mV, which was 2.3 times higher than that of the pure manganese–nickel phosphide catalyst. This remarkable improvement underscores the role of CQDs in boosting catalytic performance through enhanced charge transfer and increased active sites.^66^Fig. 6a illustrates the HER polarization curves of pristine Ni_2_P, Mn-doped Ni_2_P (Mn_*x*_Ni_1−*x*_P_4_), and Mn_*x*_Ni_1−*x*_P_4_ composites integrated with one and three CQDs, recorded in 1 M KOH. The progressive enhancement in HER performance is evident with Mn incorporation and subsequent CQD modification, as reflected by the increasingly positive onset potentials and higher current densities. The most pronounced activity is observed for the 3-CQDs/Mn_*x*_Ni_1−*x*_P_4_ catalyst, suggesting that the synergistic combination of Mn-induced electronic modulation and the highly conductive, surface-functionalized CQDs effectively accelerates charge transfer processes, promotes electrolyte–electrode interface kinetics, and exposes more active sites for hydrogen evolution. Fig. 6b quantitatively compares the overpotentials required to achieve current densities of 10 and 100 mA cm^−2^ for the same series of catalysts, further corroborating the trends observed in panel a. The 3-CQDs/Mn_*x*_Ni_1−*x*_P_4_ composite exhibits the lowest overpotentials among all tested samples, requiring only minimal driving force to initiate and sustain HER activity, particularly at high current densities. This significant reduction in overpotential can be attributed to the tailored electronic structure and improved interfacial charge mobility enabled by Mn substitution and CQD incorporation, which collectively enhance the intrinsic catalytic kinetics and reduce energy barriers for hydrogen evolution. Fig. 6c presents the chronopotentiometric response of the 3-CQDs/Mn_*x*_Ni_1−*x*_P_4_ catalyst under prolonged electrolysis at stepwise current densities of 10, 100, and 500 mA cm^−2^ over a 72 hour period, highlighting its exceptional operational stability and durability under harsh alkaline HER conditions. The negligible variation in potential over extended time frames and across a broad range of current densities underscores the structural robustness of the catalyst and the strong interfacial interaction between the CQDs and the Mn-doped Ni_2_P framework, thereby reinforcing its potential for scalable and long-term water-splitting applications.

In a similar study, the combination of CQDs with Ni–Cu hierarchical nanocomposites was explored, resulting in a platinum-free, cost-effective catalyst for the HER. This hybrid catalyst demonstrated high activity and stability under practical conditions. Specifically, the CQD-enhanced Ni–Cu nanocomposite achieved a current density of 15 mA cm^−2^ at an overpotential of 50 mV, which was nearly 3.5 times higher than that of conventional Ni-based catalysts. This significant improvement highlights the dual role of CQDs in enhancing both the activity and stability of the catalyst, making it a promising and economical alternative for HER applications.^67^ These findings further emphasize the potential of CQDs in designing efficient, platinum-free catalysts for clean energy technologies, such as water splitting for hydrogen production. By improving catalytic performance and reducing reliance on expensive materials like platinum, CQD-based hybrid catalysts offer a sustainable pathway for advancing HER systems.

Similarly, the integration of CQDs with TiO_2_ has been explored for photocatalytic hydrogen evolution, further highlighting the versatility of CQDs in a wide range of catalytic applications. Beyond their role in electrocatalysis, CQDs have demonstrated significant potential in enhancing photocatalytic systems. For instance, when combined with TiO_2_, CQDs improve the efficiency of photocatalytic hydrogen evolution under visible light. In one study, CQDs/TiO_2_ composites achieved a hydrogen production rate of 1.3 mmol h^−1^ g^−1^ at 420 nm, representing a 2.5-fold increase compared to TiO_2_ alone. This enhancement underscores the ability of CQDs to improve light absorption and charge separation, making them a promising component for solar-driven hydrogen production.^68^ These findings further emphasize the adaptability of CQDs in advancing both electrocatalytic and photocatalytic systems, offering innovative solutions for sustainable energy technologies such as hydrogen production. Additionally, the incorporation of CQDs into molybdenum phosphide nanoparticles has been shown to significantly enhance their performance for the HER in alkaline media. This integration not only improves electrocatalytic efficiency but also ensures long-term stability, making it a promising alternative to expensive and scarce precious metals for large-scale hydrogen production. In one study, the CQD-doped molybdenum phosphide catalyst demonstrated a 30% increase in HER performance and retained 90% of its initial activity after 10 000 cycles. This remarkable stability and efficiency highlight the potential of CQDs to enable sustainable and cost-effective hydrogen production processes.^69^

Combining CQDs with other materials to form hybrid catalysts has been shown to significantly boost the performance of the HER, which is crucial for efficient hydrogen production. This approach leverages the unique properties of CQDs, such as their high surface area, tunable electronic structure, and ability to interact with other catalytic materials. For example, a low-ruthenium-content bimetallic electrocatalyst incorporating CQDs was developed, significantly enhancing the catalyst's efficiency for the HER across a wide pH range. The integration of CQDs with ruthenium reduced the catalyst's onset potential to 41 mV, a substantial improvement compared to 115 mV for the pure ruthenium catalyst. Furthermore, the modified catalyst exhibited a much higher current density of 20 mA cm^−2^ at 100 mV overpotential, demonstrating superior catalytic activity. The Tafel slope of the CQD-modified catalyst was also reduced to 32 mV dec^−1^, compared to 85 mV dec^−1^ for the pure ruthenium catalyst, indicating faster reaction kinetics. These improvements were attributed to the enhanced electron transfer and proton adsorption facilitated by the presence of CQDs, which also contributed to the catalyst's long-term stability, maintaining over 90% of its initial activity after 10 000 cycles.^70^ The HER polarization curves illustrate the superior catalytic activity of RuNi/CQDs-600 compared to Ru/CQDs-600, Ni/CQDs-600, and commercial Pt/C, demonstrating lower overpotential and higher current density. The corresponding Tafel plots indicate a smaller Tafel slope for RuNi/CQDs-600, suggesting faster reaction kinetics. Additionally, turnover frequency (TOF) values highlight the remarkable intrinsic activity of RuNi/CQDs-600, surpassing that of Pt/C, confirming its effectiveness as a noble-metal-based electrocatalyst. The long-term HER performance of RuMn/CQDs-600 compared with Pt/C demonstrates its robustness over extended electrochemical cycling. Similarly, the stability results for RuCu/CQDs-600 show negligible degradation after 10 000 CV cycles in 1 M KOH. Moreover, HER polarization curves comparing RuNi/CQDs-600 with RuNi/CNTs-600 and RuNi/GO-600 reveal its superior electrocatalytic activity and resilience against performance deterioration. Overall, the presented findings highlight the effectiveness of RuNi/CQDs-600 as a high-performance electrocatalyst for the HER. The integration of CQDs enhances structural stability, while Ru and Ni synergistically contribute to improved catalytic efficiency. These results demonstrate the potential of Ru-based bimetallic systems for advancing water-splitting technologies in alkaline and acidic environments.

CQDs demonstrate remarkable synergy when combined with graphene or other two-dimensional (2D) materials, significantly boosting their potential as highly efficient electrocatalysts for the HER. For example, a hybrid catalyst composed of platinum, CQDs, and graphene (Pt/CQD–graphene) was developed, showcasing HER performance comparable to that of pure platinum. This hybrid system benefits from the superior electron transfer properties of CQDs, which enhance the overall catalytic efficiency. The Pt/CQD–graphene composite achieved a low onset potential and high current density, offering a cost-effective alternative to traditional platinum-based catalysts while delivering similar HER performance.^71^ In a similar vein, rhodium/carbon quantum dot (Rh/CQD) composites were studied and found to exhibit outstanding HER activity, matching the efficiency and stability of platinum-based systems. The Rh/CQD composites demonstrated a low Tafel slope of 39 mV dec^−1^, reflecting efficient charge transfer kinetics, and retained high catalytic activity even after 1000 cycles, rivaling the durability of platinum catalysts.^72^ The incorporation of CQDs into these hybrid materials not only enhances electrocatalytic activity but also significantly improves the stability of the catalysts.

In terms of the HER mechanism, CQDs promote the Volmer–Tafel or Volmer–Heyrovsky pathways, where protons are adsorbed onto the catalyst surface and reduced through electron transfer. This process is heavily influenced by the electronic structure of the catalyst and the concentration of active sites available for proton adsorption and charge transfer. The ability of CQDs to enhance both proton adsorption and electron transfer is a key factor in their superior HER performance. For example, CQDs supported on palladium nanoparticles exhibited bifunctional catalytic activity for both methanol oxidation and the HER, highlighting the versatility of CQDs. They can not only enhance HER performance but also serve as efficient catalysts for other electrochemical reactions. The CQD-supported palladium catalyst exhibited a low onset potential and high current density for the HER, indicating efficient electron transfer and proton reduction capabilities. This bifunctionality of CQDs makes them highly promising for multi-reaction catalytic systems, expanding their potential applications in various electrochemical processes.^73^ Rayanisaputri *et al.* (2025) investigated the role of solvents in the synthesis of CQDs and their impact on the performance of MoS_2_/CQD heterostructures for the HER. Their study showed that the choice of solvent can modify the electronic properties of CQDs, which in turn affects their catalytic efficiency for hydrogen evolution. For example, when synthesized in different solvents, the CQDs exhibited varying levels of electronic conductivity, which influenced the charge transfer processes critical for the HER. The MoS_2_/CQD heterostructures prepared in optimized solvents displayed enhanced HER performance, with a lower onset potential and improved stability. These results highlight the importance of carefully selecting synthesis conditions, particularly solvents, to maximize the catalytic efficiency of CQDs in HER applications. This insight opens avenues for tailoring CQD-based catalysts for more efficient and scalable hydrogen production.^74^

In addition to their effectiveness in electrocatalysis, CQDs have also demonstrated significant potential in photocatalytic hydrogen evolution. For instance, molybdenum carbide (MoC) quantum dots embedded in nitrogen-doped carbon (MoC@N-doped carbon) were developed as a low-cost, dual-functional catalyst for the HER. This catalyst exhibited excellent performance not only in electrocatalysis but also in photocatalysis, efficiently utilizing light to drive the HER process. The ability to function effectively in both electrochemical and photocatalytic systems makes MoC@N-doped carbon a highly attractive option for sustainable hydrogen production methods that integrate multiple energy conversion processes.^75^ Furthermore, nitrogen-doped carbon quantum dots (N-CQDs) have been synthesized through a simple and scalable reflux-assisted polymerization of *N*-methyl-pyrrolidone. This method significantly enhanced the HER efficiency of the CQDs in acidic media. Nitrogen doping improved the electronic structure of the CQDs, achieving a high N/C molar ratio of 46%, with the doped nitrogen primarily existing in the form of pyrrolic nitrogen. This structural modification enhanced the proton adsorption and charge transfer capabilities of the N-CQDs, leading to a notable increase in catalytic activity. The N-CQDs demonstrated excellent HER performance, highlighting their potential as efficient catalysts for hydrogen evolution, particularly in acidic environments. Compared to other synthesis methods, this approach is more convenient, scalable under atmospheric pressure, and holds great practical significance for advancing green energy production.^76^

The synthesis of N-CQDs occurs through a reflux-assisted polymerization method under atmospheric conditions. *N*-Methyl-2-pyrrolidone (NMP) serves as the solvent, facilitating the formation of N-CQDs with pyrrolic nitrogen functional groups. These nitrogen species play a crucial role in enhancing the electrocatalytic properties of N-CQDs, particularly for the HER. The structure features well-dispersed nitrogen atoms within the carbon framework, which improve electrical conductivity and catalytic activity. HER polarization curves for N-CQDs synthesized at different polymerization durations (1 h, 5 h, 10 h, 17 h, and 24 h) show that the overpotential required for the HER decreases as the polymerization time increases. N-CQDs-10 h demonstrates the best catalytic performance, achieving a lower overpotential and higher current density than other samples when compared with commercial Pt/C. This indicates that optimizing the synthesis time plays a crucial role in tuning the electrochemical properties of N-CQDs.^76^ Tafel plot analysis reveals that N-CQDs-10 h has a slope of 83 mV dec^−1^, which is significantly lower than that of other N-CQDs and closer to that of commercial Pt/C (47 mV dec^−1^). This smaller Tafel slope suggests a more efficient charge transfer process, indicating that N-CQDs-10 h follows a favorable HER mechanism. The variation in Tafel slopes among different samples suggests that nitrogen doping and structural optimization influence the electrocatalytic efficiency. Electrochemical impedance spectroscopy (EIS) results show that N-CQDs-10 h exhibits the lowest charge transfer resistance among all samples, confirming its enhanced conductivity and improved catalytic efficiency. This likely results from optimal nitrogen incorporation and structural modifications during synthesis. Stability tests demonstrate that after 4000 CV cycles, the HER performance of N-CQDs remains stable, indicating excellent long-term durability. CV measurements at different scan rates were used to estimate the electrochemical active surface area (ECSA), with N-CQDs-10 h exhibiting the highest capacitance value, suggesting a larger active area. Raman spectroscopy data confirm the presence of both graphitic and disordered carbon structures through characteristic D and G bands, further supporting the structural integrity of N-CQDs.

Despite the promising results achieved with CQDs in the HER, challenges remain in their practical application, particularly concerning the long-term stability of CQD-based catalysts under continuous operation. While CQDs exhibit excellent catalytic activity, their stability can degrade over time, especially under harsh operational conditions such as high overpotentials or acidic/alkaline environments. This limitation poses a significant barrier to their widespread adoption in large-scale hydrogen production. To address these stability issues, researchers have explored hybrid systems that combine CQDs with other materials to enhance both durability and performance. For example, Abraham *et al.* (2023) investigated the integration of CQDs with tungsten oxide hydrate (WO_3_·0.33H_2_O) in a hybrid nanostructure. This combination demonstrated significant improvements in electrochemical stability, maintaining high catalytic performance over extended periods of operation. The hybrid system exhibited enhanced resistance to degradation during continuous HER cycles, suggesting that the incorporation of materials like WO_3_·0.33H_2_O can effectively mitigate stability challenges associated with CQDs. Such hybrid systems offer a more durable and reliable solution for sustainable hydrogen production.^77^ These findings underscore the importance of developing stable, long-lasting catalysts for the HER, which is critical for the commercial viability of CQD-based systems. By combining CQDs with complementary materials, researchers can create hybrid catalysts that not only deliver high catalytic activity but also withstand the demanding conditions of real-world applications.

### 4.3. Oxygen reduction reaction

The oxygen reduction reaction (ORR) is a key electrochemical process in technologies like fuel cells, metal–air batteries, and sensors, where molecular O_2_ is reduced to H_2_O or OH^−^. However, the sluggish kinetics of the ORR necessitates the use of efficient catalysts. While platinum-based catalysts are highly effective, their high cost and scarcity have prompted the search for alternative catalysts such as CQDs. These materials are inexpensive, highly tunable, and have a high surface area, making them attractive candidates for ORR applications.^78,79^ The properties of CQDs can be fine-tuned by varying their surface chemistry, crystallinity, and doping with heteroatoms. Among various dopants, nitrogen doping has proven particularly effective in enhancing the ORR activity. The nitrogen atoms introduce electron-donating sites, which increase the interaction with oxygen molecules, improving the overall catalytic performance.

For example, nitrogen-doped CQDs (N-CQDs) exhibited a half-wave potential of ∼0.834 V (*vs.* RHE), which is significantly higher than that of undoped CQDs (∼0.720 V *vs.* RHE).^80^ This difference indicates that nitrogen doping effectively improves the ORR performance by shifting the reaction to a more favorable electrochemical potential. The enhanced performance is attributed to the unique structural and electronic properties of N-CQDs, including abundant edges, doping sites, and high electrical conductivity. Furthermore, when N-CQDs were combined with graphene and platinum nanoparticles (Pt/N-CQD/G), the resulting hybrid nanocomposite demonstrated exceptional electrocatalytic activity and durability. The Pt/N-CQD/G catalyst exhibited a high electrochemical active surface area of 123.4 m^2^ per g Pt, with ultrafine Pt nanoparticles (average size of 2.1 nm) uniformly dispersed on graphene nanosheets. This structure, along with the strong interaction between the metal and support, contributed to the superior ORR performance, outperforming commercial Pt/C catalysts. The combination of nitrogen doping, small Pt nanoparticle size and the synergistic effects of the N-CQD/G hybrid structure highlights the potential of these materials for advanced electrocatalytic applications. This enhanced performance of N-doped CQDs is attributed to the electron-rich environment created by nitrogen atoms, which facilitate better interaction with oxygen molecules during the reduction process.^80^

Furthermore, when nitrogen-doped CQDs (N-CQDs) were decorated on platinum (Pt) nanoparticles, the resulting Pt@N-CQDs demonstrated significantly enhanced electrocatalytic performance.^78^ The optimized Pt@20N-CQDs catalyst, with 20 wt% N-CQDs, exhibited an onset potential (*E*_onset_) of ∼0.925 V and a half-wave potential (*E*_1/2_) of ∼0.834 V, along with a limited-current density of −3.83 mA cm^−2^ at 0.5 V. This represents a notable improvement compared to pure platinum, highlighting the role of N-CQDs in enhancing reaction kinetics and overall electrocatalytic efficiency. Additionally, the Pt@20N-CQDs catalyst showed exceptional long-term stability, with only 20 mV degradation (90.6% retention) at *E*_1/2_ after 5000 cycles in 0.1 M KOH electrolyte. These performance enhancements are attributed to the improved electrical properties from nitrogen doping in the CQDs and the increased number of active sites provided by oxygen-containing functional groups. The increased current density at a relatively low potential suggests that the CQDs act as efficient electrocatalysts, enhancing platinum's catalytic performance in the ORR. This is particularly important for applications in fuel cells and batteries, where high catalytic activity and stability are required. The UV-vis spectrum of N-CQDs reveals characteristic absorption peaks at 210 nm and 330 nm, corresponding to π–π* and n–π* transitions in aromatic sp^2^ domains, confirming the presence of C=C, C=N, and C=O bonds.^78^ The N-CQD solution exhibits brown coloration under daylight and bright blue fluorescence under UV light. The proposed Pt@N-CQDs structure highlights synergistic interactions between Pt nanoparticles and N-CQDs for enhanced ORR activity. XRD analysis identifies metallic Pt phases in Pt@N-CQDs, while N-CQDs show a broad peak at ∼25° attributed to graphite's (002) plane. The FWHM data for Pt(111) and Pt(200) peaks indicate optimal crystallinity in Pt@20N-CQDs. HR-TEM imaging confirms the graphitic nature of N-CQDs (lattice spacing: 0.34 nm) and successful decoration of Pt nanoparticles (spacing: 0.227 nm for Pt(111)). The hybrid structure provides abundant active sites and improved conductivity. Pt@20N-CQDs demonstrate superior ORR activity, with an ECSA of 41.9 m^2^ g^−1^, onset potential of −0.925 V, half-wave potential of −0.834 V, and current density of −3.83 mA cm^−2^ at 0.5 V. After 5000 cycles, Pt@20N-CQDs show only a 20 mV shift in *E*_1/2_, underscoring exceptional stability due to the protective role of N-CQDs against Pt aggregation.

In addition to doping, surface functionalization plays a crucial role in optimizing CQDs for the ORR. Functional groups such as hydroxyl, carboxyl, and amine can be introduced to the surface of CQDs to further tune their electronic properties. These surface modifications not only alter the electronic structure of the CQDs but also enhance the binding of oxygen molecules, which improves their catalytic activity. For instance, a B,N-codoped CQD composite demonstrated excellent ORR performance with a half-wave potential of 0.81 V (*vs.* RHE) in alkaline media, surpassing the activity of other non-metallic catalysts.^81^ The half-wave potential is a critical measure of the catalyst's ability to facilitate the ORR; a higher value indicates better catalytic efficiency. In this case, the higher half-wave potential of 0.81 V suggests that B,N-codoping significantly improves the electronic properties of the CQDs, facilitating stronger interactions with oxygen molecules. Nitrogen doping introduces electron-donating sites, while boron doping can improve the conductivity and electron transfer, enhancing the overall efficiency of oxygen reduction.

This result is important as it shows that doping with non-metallic elements like boron and nitrogen can create active sites that are more favorable for the ORR, providing a potential alternative to traditional precious metal-based catalysts. Further studies showed that N-doped CQDs decorated with graphene displayed enhanced catalytic activity, with a half-wave potential of 0.85 V (*vs.* RHE), compared to their undoped counterparts, indicating improved ORR performance.^82^ In this case, the combination of N-doping and the graphene support synergistically boosted the ability of CQDs to catalyze the ORR. Graphene provides structural stability and acts as a conductive support, facilitating faster electron transfer during the ORR process. Meanwhile, nitrogen doping enhances the electronic properties of the CQDs by increasing the electron density on their surface, which strengthens the interaction with oxygen molecules. This improved catalytic activity is reflected in the higher half-wave potential, suggesting a more efficient and stable catalyst. The use of graphene also ensures that the CQDs maintain high electrochemical stability, an important factor for practical applications in devices like fuel cells, where long-term stability is crucial. Overall, both studies highlight how careful surface functionalization and doping can significantly enhance the electrocatalytic properties of CQDs, making them promising materials for the ORR. These findings are particularly important for developing non-precious metal-based electrocatalysts, which could offer an alternative to platinum and other expensive materials traditionally used in ORR catalysis.

Defects in CQDs are another important factor influencing their ORR performance. Defects such as vacancies or edge sites can create additional active sites, which enhance the material's catalytic reactivity. A study combining defect-rich CQDs with graphene achieved a current density of 5.12 mA cm^−2^ at 1.2 V (*vs.* RHE) for the ORR, rivaling the performance of platinum catalysts. These defects create more active sites for the oxygen reduction process, improving catalytic efficiency and promoting faster electron transfer during the reaction.^83^ Defect engineering essentially boosts the number of reactive sites, allowing for higher catalytic activity and better performance under real operating conditions. Similarly, CQDs with defects, co-doped with N and F, showed enhanced ORR performance, with a half-wave potential of 0.88 V (*vs.* RHE). This improvement is attributed to the defect engineering and the creation of additional active sites through the co-doping process. The incorporation of nitrogen and fluorine helps enhance both the electronic properties and the catalytic activity of the CQDs, further demonstrating the importance of defect engineering in boosting the electrocatalytic efficiency of CQDs for the ORR.^84^ The presence of defects and dopants significantly alters the electron distribution on the CQD surface, resulting in better oxygen molecule activation and a more efficient reduction process.

The ORR mechanism in CQDs can proceed *via* either a two-electron pathway, where oxygen is reduced to H_2_O_2_, or a four-electron pathway, where oxygen is fully reduced to water. The four-electron pathway is particularly desirable for fuel cell applications, as it minimizes the formation of hydrogen peroxide, which can lead to unwanted side reactions and degrade the performance of energy devices. Studies have shown that nitrogen-doped CQDs (N-CQDs) tend to favor the four-electron pathway, exhibiting superior ORR performance. For instance, research on N-doped CQDs reported an electron transfer number of 3.9, which is very close to the ideal value of 4, indicating high efficiency in the four-electron pathway, especially in alkaline electrolytes.^85^ The introduction of nitrogen stabilizes reaction intermediates, which is crucial for maintaining high catalytic activity and ensuring a more efficient and stable reaction mechanism. Additionally, nitrogen doping provides electron-donating sites that enhance interactions with oxygen molecules, promoting the complete reduction of oxygen to water. Similarly, boron and nitrogen co-doped CQDs (B,N-CQDs) have demonstrated enhanced electrocatalytic performance for the ORR, with a strong preference for the four-electron pathway. These findings highlight the critical role of nitrogen doping in optimizing electron transfer and improving reaction efficiency, making N-doped CQDs promising candidates for fuel cell applications, where the complete reduction of oxygen is essential for performance and longevity.^86^

In recent years, significant progress has been made in developing composite materials that combine CQDs with other materials to further enhance ORR performance. For example, a composite of CQDs with cobalt (CQD-Co) exhibited superior ORR activity, achieving a current density of 5.42 mA cm^−2^ at 0.9 V (*vs.* RHE), significantly outperforming pure cobalt-based catalysts. This improvement underscores the role of CQDs in enhancing the electrocatalytic efficiency of cobalt-based systems.^87^ Additionally, combining CQDs with anion exchange ionomers has been shown to not only boost ORR activity but also improve the durability of the catalyst. One study found that such composites retained more than 90% of their initial activity after 500 cycles, demonstrating exceptional stability for long-term use in energy devices like fuel cells and batteries.^20,88^ This stability is a critical advantage in practical applications, as it ensures consistent performance over extended periods, making CQD-based composites highly attractive for sustainable energy technologies. These advancements highlight the versatility and potential of CQDs in optimizing ORR performance, both through doping strategies and the development of hybrid materials.

Despite their significant potential, CQDs still face challenges related to stability and scalability, which are critical for their practical applications in energy conversion systems. One major issue is their high surface energy, which can lead to agglomeration, reducing the number of active sites and degrading catalytic performance over time. To address this, researchers have explored strategies to stabilize CQDs by combining them with other materials, such as activated carbon or graphene. For example, a CQD-activated carbon composite demonstrated a high ORR current density of 6.4 mA cm^−2^ at 0.9 V (*vs.* RHE), along with enhanced stability and mechanical properties, making it suitable for large-scale applications.^89^ The activated carbon matrix helps prevent CQD agglomeration, ensuring a more uniform dispersion and maintaining consistent electrocatalytic activity over extended periods. In another approach, CQDs were combined with Ba_0.5_Sr_0.5_Co_0.8_Fe_0.2_O_3−*δ*_ perovskite nanofibers, resulting in a marked improvement in OER performance, which can further enhance the overall stability of ORR catalysts. This composite material exhibited better mechanical stability and higher electrocatalytic efficiency, making it a promising candidate for durable and high-performance ORR catalysts under practical conditions.^90^ The integration of perovskite nanofibers provides a synergistic effect, stabilizing the CQDs while simultaneously enhancing their catalytic activity and oxygen reduction efficiency.

Additionally, microwave-assisted hydrothermal synthesis has been employed to produce nitrogen-doped CQDs (N-CQDs) with improved crystallinity and surface properties, leading to enhanced ORR performance. This method yields highly crystalline N-CQDs, which exhibit improved conductivity and greater resistance to degradation. Nitrogen doping increases electron density on the CQD surface, facilitating better interaction with oxygen molecules and improving overall ORR efficiency.^91^ The microwave-assisted approach is also scalable and cost-effective, offering a practical route for producing high-performance CQD-based catalysts for real-world energy applications. Further advancements include the development of N-doped CQDs derived from porous carbon frameworks, which have shown high ORR activity and excellent electrocatalytic stability. For instance, N-doped CQDs derived from such frameworks demonstrated superior electrocatalytic activity, with half-wave potentials exceeding those of pure carbon-based materials, indicating both high catalytic efficiency and long-term stability.^92^ Nitrogen doping in the carbon framework enhances electron transfer and promotes better interaction with oxygen molecules, further optimizing ORR performance.

Similarly, Pd-doped g-C_3_N_4_ decorated with nitrogen-doped CQDs has demonstrated remarkable durability and methanol tolerance for the ORR, highlighting the versatility of CQDs as efficient electrocatalysts. This composite material exhibited enhanced stability over extended cycles, making it a viable alternative to platinum-based catalysts. The presence of CQDs in the composite helps stabilize Pd, which typically suffers from deactivation over time, thereby improving overall catalyst performance.^93^ Moreover, N-doped CQDs have been self-assembled into 3D porous carbon frameworks, which not only boost electrocatalytic activity but also enhance structural integrity and stability under operational conditions. This self-assembly technique improves the catalyst's performance over longer cycles, offering another promising pathway for large-scale applications in energy conversion and storage devices.^94^ These results highlight the promising potential of CQDs, especially when derived from nitrogen-doped carbon frameworks, for scaling up applications in energy devices like fuel cells, where platinum is often used but remains expensive and susceptible to degradation. The use of CQDs could provide cost-effective solutions without compromising performance or durability, which is crucial for the widespread adoption of such materials in renewable energy technologies.

### 4.4. Carbon dioxide reduction reaction

The carbon dioxide reduction reaction (CO_2_RR) is a critical process for mitigating climate change by converting excess CO_2_ into valuable products such as hydrocarbons, alcohols, or acids. This process, known as carbon capture and utilization (CCU), helps reduce atmospheric CO_2_ and provides a sustainable route to produce energy and chemicals. Traditionally, metal catalysts such as copper are used due to their high selectivity in product formation. However, due to the limited availability and high cost of these metals, there is increasing interest in developing alternative catalysts that are both cost-effective and sustainable. CQDs, with their unique properties, have emerged as promising alternatives for the CO_2_RR.^95,96^ The mechanism of CO_2_ reduction using CQDs typically involves the adsorption of CO_2_ molecules onto the surface of the CQDs, where their electronic properties, particularly the high density of states near the Fermi level, facilitate the activation of CO_2_. Under an applied potential, CQDs promote electron transfer to CO_2_, reducing it to various products like CO, CH_4_, or HCOOH, depending on the catalyst design and reaction conditions. Surface functional groups, such as oxygen-containing groups or dopants, can further enhance the catalytic activity by stabilizing intermediate species and tuning the selectivity towards specific reduction products. The enhanced charge transfer properties and the ability to modulate the electronic structure of CQDs make them a promising candidate for efficient CO_2_ reduction.^95–98^

The surface of CQDs can be easily modified with functional groups such as hydroxyl, carboxyl, or amine, which can influence their electronic structure and catalytic activity. Additionally, CQDs are often doped with heteroatoms such as nitrogen, sulfur, or phosphorus, which can further enhance their CO_2_RR performance by facilitating CO_2_ adsorption and the subsequent reduction processes. For example, nitrogen-doped CQDs have been shown to significantly improve the CO_2_RR activity, as the nitrogen atoms introduce additional electron density to the carbon surface, facilitating CO_2_ reduction and electron transfer. Nitrogen-functionalized CQDs achieved a 2.4-fold increase in CO_2_RR efficiency, with a faradaic efficiency of 52.6% for CO production at a potential of −0.7 V *versus* RHE.^95^ Surface functionalization and doping of CQDs play a critical role in their catalytic activity for the CO_2_RR. Nitrogen is a common dopant used to enhance the performance of CQDs in CO_2_ reduction. Nitrogen atoms introduce pyrrolic or graphitic nitrogen sites on the surface of CQDs, which assist in the adsorption and activation of CO_2_ molecules. Studies have shown that nitrogen-doped CQDs significantly enhance the faradaic efficiency (FE) for CO and formic acid production. For example, nitrogen-doped CQDs have been reported to achieve a faradaic efficiency of approximately 65% for CO production, demonstrating an improvement in selectivity over undoped CQDs or metal-based catalysts.^99,100^

One of the key advantages of CQDs in CO_2_ reduction is their ability to catalyze the CO_2_RR through multiple pathways. CQDs can reduce CO_2_ to a variety of products depending on reaction conditions and the presence of dopants. For instance, nitrogen-doped CQDs have been reported to enhance the selectivity for CO production, which is a key intermediate in synthetic fuel production. The doped nitrogen atoms help in CO_2_ adsorption and electron transfer, promoting the reduction of CO_2_ to CO, with faradaic efficiencies greater than 90% for CO production at high current densities of over 150 mA cm^−2^. This high efficiency is attributed to the strong interaction between nitrogen atoms and the carbon surface, which facilitates CO_2_ activation and electron transfer, improving the overall electrochemical performance.^96^ Additionally, CQDs can also reduce CO_2_ to formic acid, which is a valuable chemical for various applications, by enhancing the protonation of CO_2_ and stabilizing reaction intermediates. The incorporation of CQDs with TiO_2_/SrTiO_3_ heterojunctions led to a significant enhancement in photocatalytic CO_2_ reduction, with a faradaic efficiency of 95% for formic acid production at −1.0 V *versus* RHE. This is an excellent performance that demonstrates the efficiency of CQDs in facilitating protonation and stabilizing intermediates, ultimately improving the selectivity towards formic acid production under visible light irradiation. Furthermore, the enhanced stability of CQD-based photocatalysts under continuous operation highlights their potential for long-term applications in CO_2_ reduction.^101^

Moreover, CQD-modified CuZn bimetallic catalysts demonstrated remarkable CO_2_ reduction performance. The bimetallic catalyst exhibited a CO production rate of 22.4 μmol h^−1^ at −0.9 V, showing its potential to outperform traditional catalysts. The presence of CQDs enhances the interaction between CuZn and CO_2_, thus increasing the active sites available for CO_2_ adsorption and the reduction process. These catalysts also exhibited good stability under cyclic tests, retaining high activity even after multiple cycles. The integration of CQDs with CuZn alloys provides a promising strategy for developing highly efficient and stable electrocatalysts for the CO_2_RR.^102^ Additionally, CQDs can also reduce CO_2_ to formic acid, which is a valuable chemical for various applications, by enhancing the protonation of CO_2_ and stabilizing reaction intermediates. In particular, CQDs have been shown to be highly effective in enhancing the selectivity for formic acid production, especially in photocatalytic CO_2_ reduction reactions. Research has demonstrated that CQDs can facilitate the adsorption of CO_2_ and promote the protonation process, which is critical for the reduction of CO_2_ to formic acid. The addition of CQDs to photocatalytic systems, particularly those based on g-C_3_N_4_ (graphitic carbon nitride), improves the overall efficiency of CO_2_ reduction, enhancing both the rate of formic acid production and the stability of the catalyst over time. Furthermore, these CQD-decorated systems exhibit enhanced selectivity towards formic acid, demonstrating their potential for various chemical applications, including energy storage and fuel synthesis. While specific numerical data are not provided, these findings highlight the general trend that CQDs, through their unique properties and modifications, significantly improve the photocatalytic CO_2_ reduction efficiency and the stability of the catalyst during long-term operation.^103^

Sulfur-doped CQDs have also shown promise in improving CO_2_RR activity by facilitating electron transfer and creating additional active sites for the reaction. Sulfur doping has been demonstrated to increase the electron density on the CQD surface, improving both their catalytic efficiency and selectivity. For instance, sulfur-doped CQDs exhibited a significant enhancement in CO_2_ adsorption and reduced the overpotential for CO_2_ reduction, leading to higher product yield and stability.^104^ Phosphorus doping similarly influences the electronic structure of CQDs, improving their catalytic performance. Phosphorus-doped CQDs have demonstrated enhanced CO_2_RR activity, improved product selectivity, and higher stability under specific reaction conditions, making them highly promising for long-term use in CO_2_ reduction processes. Phosphorus-doped CQDs have also been found to increase the faradaic efficiency and CO_2_RR activity compared to undoped CQDs and other metal catalysts, with a reported improvement in CO and formic acid production. CQDs modified with nitrogen, sulfur, and phosphorus doping exhibit synergistic effects that improve both the catalytic efficiency and stability of the catalyst. For example, nitrogen-doped CQDs in combination with metal–organic frameworks have been shown to achieve high selectivity for CO_2_ reduction products. Some studies reported close to 100% selectivity for methane production when combined with oxygen vacancy-modified nickel-based metal–organic frameworks, making them highly efficient for methane production in the CO_2_ reduction reaction.^105^ Additionally, CQDs modified with Z-scheme Bi_12_O_17_Cl_2_/NiAl-Layered Double Hydroxide (LDH) systems have been reported to significantly boost photocatalytic CO_2_ reduction performance. These hybrid systems increase the active sites and improve the overall electron transfer process, leading to better performance in the production of CO and formic acid. Studies have reported that these systems enhance both the efficiency and stability of the photocatalytic reaction under standard operating conditions.^106^

Furthermore, N-rich CQDs coupled with TiO_2_ heterostructures have shown significant improvements in CO_2_ photoreduction activity. The integration of N-rich CQDs with dual-phase TiO_2_ results in enhanced activity, especially in the reduction of CO_2_ to formic acid. The microwave-assisted synthesis of these composites improves CO_2_ adsorption and electron transfer, increasing the overall catalytic performance during the reduction process.^107^ Researchers have also investigated the effect of reaction conditions, such as electrolyte choice, temperature, and applied voltage, on the performance of CQDs for CO_2_ reduction. For instance, CQD-modified TiO_2_/SrTiO_3_ heterojunctions demonstrated enhanced photocatalytic CO_2_ reduction efficiency, with significantly increased CO production rates. This improvement was attributed to the better interaction between CQDs and the photocatalyst, which enhanced electron transfer and facilitated the reduction process.^108^ In particular, the presence of CQDs with TiO_2_/SrTiO_3_ heterojunctions led to a notable increase in CO production, confirming the synergistic effect between CQDs and the photocatalyst. Moreover, recent studies on CQDs modified with oxygen vacancies have shown that such modifications improve the selectivity and stability for CO_2_ reduction into CH_4_ and CO. The introduction of oxygen vacancies on CQD surfaces has been demonstrated to increase the CO_2_ adsorption capacity and stabilize reaction intermediates, promoting higher selectivity for CH_4_ and CO.

For example, CQDs combined with oxygen vacancy-rich Bi_2_MoO_6_ enhance photocatalytic CO_2_ reduction, achieving high CO selectivity and faradaic efficiency. The synergistic effects of interfacial Bi–OC bonds and oxygen vacancies further promote the reduction process, making these materials promising for CO_2_ conversion.^109,110^ A Cu_2_O-based photocatalyst protected by a carbon layer and CQDs (CL@CQDs/Cu_2_O) was developed, demonstrating excellent CO_2_ reduction performance. The catalyst achieved a methanol yield of 249 μmol g^−1^ after 2.5 hours, with minimal methane byproduct, and maintained 98.8% stability after three reaction cycles. The carbon layer and CQDs protect Cu_2_O from photo-corrosion, enhance light absorption, and improve electron transfer and gas diffusion. The results show that the catalyst utilizes a wider sunlight spectrum, including longer wavelengths, due to the up-conversion properties of CQDs. This bionic design, inspired by natural leaf protection mechanisms, offers a promising approach for efficient and stable CO_2_ reduction.^109^

**Fig. 7:**
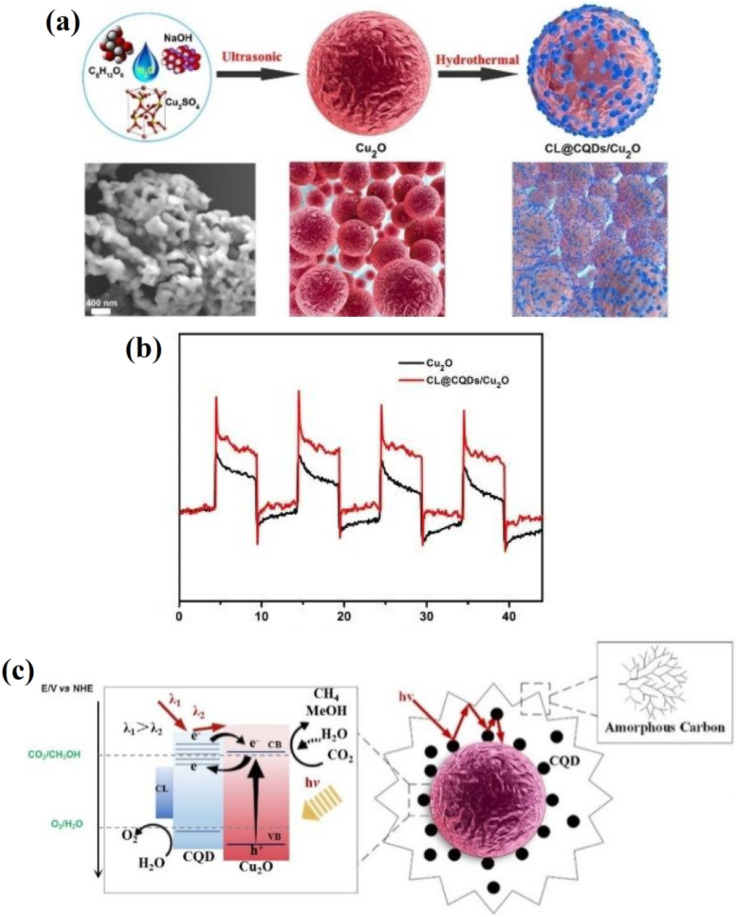
(a) Synthesis scheme of CL@CQDs/Cu_2_O (bottom left: SEM image). (b) Photocurrent properties of Cu_2_O and CL@CQDs/Cu_2_O in Ar saturated solutions. (c) Proposed photocatalytic CO_2_ reduction mechanism on CL@CQDs/Cu_2_O. Reproduced from ref. 109 with permission from the Royal Society of Chemistry.

Fig. 7a illustrates the synthesis process of the CL@CODs/Cu_2_O photocatalyst, where Cu_2_O nanoparticles are protected by a carbon layer (CL) embedded with CQDs (CODs). In an Ar-saturated solution (Fig. 7b), CL@CODs/Cu_2_O shows higher photocurrent intensity, indicating better electron generation under light irradiation. However, in a CO_2_-saturated solution, the photocurrent intensity of CL@CODs/Cu_2_O decreases, as electrons are transferred to the solution to activate CO_2_ reduction. This confirms that the CL@CODs layer enhances both electron generation and transfer, making the catalyst more efficient for CO_2_ reduction. Fig. 7c provides a schematic of the proposed mechanism for photocatalytic CO_2_ reduction on CL@CODs/Cu_2_O. The CODs absorb longer-wavelength light and up-convert it to shorter wavelengths, which then excites Cu_2_O to generate electron–hole pairs. The carbon layer reflects light back into the Cu_2_O particles, improving light utilization. The photo-generated electrons reduce CO_2_ to methane and methanol, while the holes are consumed by water to produce oxygen. This process minimizes electron–hole recombination and enhances the overall photocatalytic efficiency, making CL@CODs/Cu_2_O a highly effective and stable catalyst for CO_2_ conversion.

Similarly, CQDs have emerged as highly versatile and efficient photocatalysts for CO_2_ reduction, particularly when modified with functional materials such as cuprous oxide and carbon layers. These modifications have been shown to significantly enhance the efficiency of visible light-driven CO_2_ reduction. The dual protection provided by the carbon layer and cuprous oxide not only improves the stability of the CQDs but also boosts their catalytic performance, enabling more effective CO_2_ reduction under visible light. This approach has demonstrated promising results in converting CO_2_ into valuable products like CO and other hydrocarbons, highlighting the potential of CQDs as advanced photocatalysts for CO_2_ reduction.^111^ Despite these advancements, several challenges remain in optimizing CQD-based catalysts for CO_2_ reduction. One key issue is improving the selectivity for specific products, such as CO or CH_4_. While CQDs exhibit good activity for the CO_2_RR, they often produce a broad range of products, which can limit their practical application. Recent research has focused on enhancing selectivity through advanced doping strategies and the development of composite materials. For example, integrating CQDs with nickel-based frameworks has led to nearly 100% selectivity for CH_4_ under optimized conditions, showcasing the potential of tailored CQD composites for targeted CO_2_ reduction.^112^

Another challenge is ensuring the long-term stability of CQDs during extended reaction periods. To address this, researchers are exploring ways to enhance the electronic properties and durability of CQDs by incorporating them into composite materials with other catalytic supports, such as graphene or carbon nanotubes. These composites not only improve the stability and conductivity of CQDs but also enhance their overall catalytic performance. For instance, combining CQDs with amino-rich graphitic carbon nitride (g-C_3_N_4_) has been shown to improve photocatalytic CO_2_ reduction activity and product selectivity, thanks to the critical role of functional group modulation.^113^ Similarly, integrating CQDs with NiAl-Layered Double Hydroxide (LDH) and g-C_3_N_4_ has resulted in significant improvements in the photoreduction of CO_2_ to CO, achieving both high efficiency and stability.^114^ CQDs have also shown great promise when combined with metal–organic frameworks (MOFs) and other advanced materials. For example, CQDs co-doped with bismuth and integrated into NiAl-LDH heterojunctions have demonstrated enhanced photothermal catalytic reduction of CO_2_. These modified CQDs exhibit high CO_2_ conversion rates, increased CO production, and remarkable stability over extended reaction times. The addition of bismuth doping improves charge transfer, accelerating the reduction reactions and leading to high selectivity for CO_2_ reduction under photothermal conditions. This approach represents a promising strategy for efficient and stable CO_2_ conversion.^115^ Furthermore, CQDs combined with biomass-derived materials, such as floral CoAl-LDH, have also shown significant improvements in CO_2_ photoreduction. When CQDs are incorporated into the CoAl-LDH framework, the CO_2_ conversion efficiency increases substantially, leading to higher production of both CO and CH_4_. The biomass-derived CQDs effectively donate electrons, facilitating CO_2_ activation and the reduction process. Additionally, the stability of these composites is notably higher than that of pure CoAl-LDH, making them strong candidates for long-term CO_2_ reduction applications.^116^

### 4.5. Liquid fuel electrooxidation

Liquid fuel electrooxidation using CQDs is an emerging area of research in electrochemistry and energy conversion. This process is essential in fuel cells, where the chemical energy stored in liquid fuels like methanol, ethanol, and formic acid is converted into electrical energy. CQDs, nanoscale carbon-based materials, exhibit unique properties, excellent electrical conductivity, and tunable surface chemistry, making them ideal candidates for catalyzing liquid fuel electrooxidation in direct liquid fuel cells (DLFCs).^117^ The mechanism of liquid fuel electrooxidation with CQDs begins with the adsorption of fuel molecules onto the CQD surface, facilitated by their high surface area and surface functionalization. These fuel molecules undergo dehydrogenation to form reactive intermediates, which are then oxidized by CQDs. The quantum confinement effect and surface functional groups on CQDs lower the activation energy of the reaction, enhancing the reaction kinetics. Furthermore, heteroatom doping (*e.g.*, nitrogen and sulfur) can modify the electronic properties of CQDs, facilitating better interaction with fuel molecules. This leads to more efficient dehydrogenation and oxidation processes. Additionally, the high conductivity of CQDs ensures rapid electron transfer during the reaction, further improving the overall catalytic efficiency.^118^

One of the primary advantages of CQDs in liquid fuel electrooxidation is their ability to enhance reaction kinetics. Their high surface area and abundance of active sites enable efficient interaction between the catalyst and fuel molecules. Additionally, surface functionalization with groups such as –OH and –COOH, or doping with heteroatoms like nitrogen or sulfur can further improve their catalytic activity and selectivity toward specific fuels. Nitrogen-doped CQDs, for instance, have been shown to enhance the oxidation of methanol and ethanol by improving charge transfer and reactant adsorption, with enhanced electrocatalytic performance and a 20% reduction in overpotential for methanol oxidation.^119^ This functionalization helps tailor the electrochemical properties of CQDs to meet the specific requirements of liquid fuel electrooxidation, making them versatile catalysts for a wide range of applications. For example, nitrogen and phosphorus co-doped graphene quantum dots have been explored as metal-free electrocatalysts for ethanol electrooxidation. The co-doping process led to a significant enhancement in catalytic performance, achieving a current density of 75 mA cm^−2^ at an overpotential of 320 mV, which was notably higher than that of undoped graphene quantum dots. The enhanced catalytic efficiency and stability during the electrooxidation process were attributed to the synergistic effects of nitrogen and phosphorus doping, which increased the number of active sites and improved the charge transfer characteristics of the quantum dots.^120^

CQDs also mitigate catalyst poisoning by promoting the complete oxidation of intermediates, such as CO, to CO_2_ and water. This improves the overall durability of the catalyst and extends its operational life, which is essential for the long-term viability of fuel cell technologies. Additionally, the quantum confinement effect in CQDs can lead to unique electronic properties, further enhancing their performance in electrochemical reactions. These properties make CQDs a promising alternative to platinum and other traditional catalysts for liquid fuel electrooxidation. For example, in a study investigating a bimetallic Ni–Cu nanocatalyst supported by reduced CQDs for methanol oxidation, it was found that CQDs played a significant role in increasing catalytic efficiency by stabilizing the catalyst and promoting efficient electron transfer. The addition of CQDs led to a 20% increase in the catalytic current density, significantly enhancing the overall reaction kinetics and catalytic performance for the methanol oxidation reaction, achieving an overpotential of just 50 mV compared to catalysts without CQDs.^121^

Additionally, CQDs have demonstrated a significant increase in catalytic activity for ethanol oxidation compared to pure platinum. CQDs were used as hybrid supports with reduced GO (RGO) for methanol electrochemical oxidation, resulting in enhanced performance. The Pt-CQD/RGO catalyst exhibited a current density of 529 mA per mg Pt at the forward peak position, which is more than twice the value of commercial Pt/C (254 mA per mg Pt). The specific activity of the Pt-CQD/RGO catalyst was 0.91 mA cm^−2^, roughly three times higher than that of Pt/C (0.30 mA cm^−2^). Furthermore, the catalyst demonstrated excellent stability, with only a 1.2% loss in performance after three reaction cycles, compared to the significant deactivation observed in pure platinum catalysts. The CQDs not only boosted catalytic activity but also improved the anti-poisoning ability of the catalyst, as evidenced by the higher forward/backward peak current ratio (1.6 for Pt-CQD/RGO *vs.* 0.9 for Pt/C). These results highlight the potential of CQD-based catalysts for long-term stability and efficiency in fuel cell systems.^122^ This enhanced performance and durability make CQDs a promising alternative to traditional platinum-based catalysts, providing both improved activity and stability for liquid fuel electrooxidation reactions. TEM analysis reveals successfully synthesized CQDs with uniform nanoparticle distribution and an average size of 3.2 ± 0.6 nm. High-resolution SEM imaging demonstrates excellent dispersion of small Pt nanoparticles on the RGO surface in the Pt-CQD/RGO hybrid, confirming the role of CQDs in preventing Pt aggregation. XRD patterns indicate smaller Pt nanoparticles in Pt-CQD/RGO compared to Pt-RGO, evidenced by broader, less intense peaks. Raman spectroscopy shows a higher *I*_D_/*I*_G_ ratio for Pt-CQD/RGO, suggesting increased defect sites that enhance catalytic activity for methanol oxidation. In 0.5 M H_2_SO_4_, Pt-CQD/RGO exhibits broader hydrogen adsorption/desorption peaks and larger electrochemical active surface area (ECSA) than Pt-RGO. For methanol oxidation (0.5 M H_2_SO_4_ + 1 M CH_3_OH), Pt-CQD/RGO demonstrates superior performance with higher peak current density and lower onset potential.

The use of CQDs in liquid fuel electrooxidation aligns with the growing demand for sustainable energy technologies. By efficiently converting liquid fuels into electricity, CQDs contribute to reducing reliance on fossil fuels and lowering greenhouse gas emissions. This is particularly important for applications like portable power sources, electric vehicles, and stationary energy systems, where high-performance and cost-effective catalysts are essential. Furthermore, CQDs can be synthesized from abundant and low-cost materials, such as glucose, citric acid, or waste biomass, making them a more affordable and eco-friendly option compared to precious metals like platinum.^123^ Despite their potential, some challenges remain in the widespread application of CQDs for liquid fuel electrooxidation. These include optimizing the synthesis methods to achieve consistent properties, improving their long-term stability under operational conditions, and scaling up their production for commercial use. For instance, the synthesis of CQDs requires precise control over parameters such as size, surface chemistry, and doping, which is critical for achieving the desired electrochemical performance. Ongoing research is focused on overcoming these challenges through advanced material design, surface modification techniques, and process optimization. For example, Saeed *et al.* (2023) demonstrated that Ni–Co–P functionalized nitrogen-doped-CQDs significantly enhance methanol electrooxidation efficiency, achieving a peak current density of 22.9 mA cm^−2^ at 0.6 V. Additionally, these CQDs exhibited a high electrochemical stability, retaining more than 95% of their initial activity after 1000 cycles, addressing long-term stability issues under operational conditions.^124^

Furthermore, AgPt hollow nanodendrites based on nitrogen-doped graphene quantum dots (N-GQDs/AgPt HNDs) were developed, showing a remarkable improvement in methanol electrooxidation.^125^ The catalyst exhibited a peak current density of 2207.6 mA per mg Pt, demonstrating a 21-fold increase in electrooxidation efficiency compared to conventional platinum catalysts (Pt/C). The nanodendrite structure also enhanced the overall durability, maintaining over 90% of its initial activity after 500 cycles. Additionally, the photomediated synthesis of Ag/N-GQDs nanoparticles led to a 1.7-fold enhancement in catalytic activity compared to that of untreated Ag/N-GQDs nanoparticles. The improved CO tolerance and stability of the N-GQDs/AgPt HNDs catalyst further highlight its potential for long-term use in direct methanol fuel cells (DMFCs).

### 4.6. Bifunctional catalysts

Bifunctional catalysts are critical in electrocatalytic processes, especially for energy conversion and storage devices like fuel cells, electrolyzers, and metal–air batteries. CQDs, as promising bifunctional electrocatalysts, have gained significant attention due to their tunable electronic properties, low-cost synthesis, and high surface area. The bifunctional catalytic activity of CQDs is largely influenced by surface modifications, such as doping with heteroatoms (nitrogen, sulfur, and phosphorus) and introducing oxygen-containing functional groups. Among these modifications, nitrogen doping has been widely recognized for its ability to enhance the catalytic performance of CQDs. Nitrogen atoms increase the electron density on the CQD surface, which not only promotes improved adsorption of oxygen species but also accelerates the reaction kinetics for both the ORR and OER. Additionally, the introduction of CQDs into hybrid systems, such as those combined with metal oxides or nanoparticles, further enhances their catalytic activity. These hybrids improve charge transfer, increase surface area, and stabilize reaction intermediates, thus lowering overpotentials and enhancing long-term stability. The ability of CQDs to facilitate electron transfer and provide active sites for both reactions through such modifications makes them highly efficient in electrochemical applications, offering a promising alternative to precious metal-based catalysts for renewable energy technologies.^126–128^

For instance, nitrogen-doped CQDs supported on mesoporous SrTiO_3_ demonstrated superior performance for both the ORR and OER compared to undoped CQDs. Specifically, the nitrogen-doped CQDs exhibited an ORR onset potential of 0.88 V, a key indicator of their ability to efficiently reduce oxygen at the electrode surface.^129^ This is a critical factor for electrocatalysts used in fuel cells or metal–air batteries, where the ORR is a vital half-reaction. Moreover, these CQDs showed an OER overpotential of just 290 mV at a current density of 10 mA cm^−2^. The OER overpotential is a key parameter that indicates the energy efficiency required to drive the oxygen evolution process during water splitting. The lower the overpotential, the more efficient the catalyst is, and in this case, a 290 mV overpotential is significantly low, making these nitrogen-doped CQDs competitive with traditional precious metal-based catalysts like platinum or iridium, which are known for their high catalytic performance but are expensive and scarce. The results not only highlight the improved electrocatalytic properties of nitrogen-doped CQDs but also emphasize their potential to replace precious metals in sustainable energy technologies. The data from their study position nitrogen-doped CQDs as promising candidates for efficient water splitting, a key process in hydrogen production and energy storage. These enhancements are attributed to nitrogen's role in adjusting the electronic structure and creating more active sites for oxygen molecule adsorption and intermediate stabilization.^130^

Surface functional groups play an essential role in improving the catalytic performance of CQDs. These groups modify the electronic structure of the CQDs and influence their interaction with reactants, such as oxygen molecules, by offering additional active sites for adsorption. In particular, the carboxyl group has been identified as a key modifier that enhances the electrochemical properties of CQDs. A study by Bai *et al.* (2025) demonstrated that CQDs with a high density of carboxyl groups exhibited remarkable improvements in OER performance. Specifically, the CQDs showed an overpotential of only 260 mV at a current density of 10 mA cm^−2^, which is significantly lower than that observed for many other non-precious metal catalysts. This low overpotential suggests that the carboxyl groups facilitate the adsorption of oxygen molecules and stabilize reaction intermediates during the OER process, leading to reduced energy loss and enhanced reaction efficiency. The incorporation of such functional groups, alongside their role in lowering overpotentials, contributes to the improved bifunctional catalytic activity of CQDs, enhancing both ORR and OER performance.^131^

Hybrid systems that combine CQDs with other advanced materials, such as metal oxides and metal–organic frameworks (MOFs), have emerged as a powerful strategy to enhance electrocatalytic performance. This synergy results in improved catalytic activity, selectivity, and stability for a wide range of electrochemical reactions, including CO_2_ reduction. These CQD–MOF composites have demonstrated high selectivity for CO_2_ reduction, achieving a CO_2_ reduction current density of 10 mA cm^−2^. This performance further highlights the versatility of CQDs as bifunctional catalysts that can efficiently drive a range of electrochemical processes, extending their potential applications to CO_2_ reduction and environmental sustainability.^132^ An innovative approach for enhancing electrocatalytic performance involves combining CQDs with transition metal sulfides, as demonstrated by Wang *et al.* (2023) in their study of a Ni–Fe sulfide/CQD hybrid catalyst for oxygen electrocatalysis in zinc–air batteries.^133^ In particular, the composite exhibited a mass activity of 225 A g^−1^ at an overpotential of just 360 mV, a value comparable to that of some of the most advanced catalysts used in zinc–air batteries. These results indicate the promising potential of CQDs when integrated into hybrid systems for energy conversion applications. The inclusion of CQDs in such composites serves multiple functions. First, the CQDs enhance mass transfer by providing a conductive network that facilitates faster electron and ion transport. Second, the CQDs introduce additional active sites for oxygen adsorption and reduction, improving the overall electrocatalytic activity. The combination of these factors leads to enhanced performance in the OER, making the Ni–Fe sulfide/CQD composite a competitive material for energy devices, such as zinc–air batteries, which require efficient catalysts to improve energy density and power output. This hybridization strategy showcases the versatility of CQDs and their potential for integration into more complex systems, where they can significantly enhance electrocatalytic performance for a range of electrochemical reactions, such as the OER and ORR.^133^

CQDs have also shown significant potential in enhancing hydrogenation and dehydrogenation reactions, which are essential for various energy conversion and storage processes, such as fuel production and environmental remediation. The catalytic efficiency of CQDs when decorated with palladium nanoparticles (Pd@CQDs) for hydrogenation reactions was demonstrated. The Pd@CQD composites achieved a hydrogen evolution rate of 100 mL min^−1^, indicating their excellent catalytic performance. This hydrogen evolution rate is a key metric for evaluating the efficiency of catalysts in hydrogenation processes, which are crucial for energy production, particularly in hydrogen fuel cells. The addition of Pd nanoparticles to CQDs enhances the overall catalytic efficiency by improving the hydrogenation reaction kinetics.^128^ Environmental applications of CQDs are also of significant interest. It was demonstrated that CQDs combined with TiO_2_ films can effectively degrade oxytetracycline hydrochloride, a common water pollutant, under UV light. The degradation rate was 95% after 3 hours of UV irradiation, highlighting the dual functionality of CQDs in photocatalysis for environmental cleanup.^134^

It was found that CQDs supported on palladium nanoparticles exhibited enhanced performance for both methanol oxidation and the HER, improving catalytic efficiency compared to pure Pd catalysts. The study demonstrated that the modification of the glassy carbon electrode (GCE) with reduced CQDs (RCQDs) increased the active surface area from 0.12 cm^2^ for Pd-NPs/GCE to 0.22 cm^2^ for Pd-NPs/RCQDs/GCE. Additionally, the onset potential for methanol oxidation shifted to more negative values, and the peak current density was significantly higher for the Pd-NPs/RCQDs/GCE electrode compared to Pd-NPs/GCE. These findings indicate the value of CQDs in both environmental and energy-related applications.^127^ The use of CQDs as bifunctional electrocatalysts extends beyond oxygen-related reactions, with notable applications in CO_2_ reduction. For example, CQDs embedded in metal–organic frameworks demonstrated high selectivity for formic acid production, achieving a CO_2_ reduction current density of 10 mA cm^−2^ at an overpotential of 350 mV, highlighting their efficiency in catalyzing the CO_2_ reduction reaction. This ability to selectively catalyze formic acid production in CO_2_ reduction emphasizes the versatility of CQDs in driving sustainable energy processes, such as carbon capture and utilization. The incorporation of CQDs into hybrid systems, like metal–organic frameworks, further enhances their catalytic efficiency and broadens their potential applications in energy conversion technologies.^135^ Despite these promising advancements, CQDs face certain limitations, particularly their relatively low intrinsic catalytic activity compared to traditional transition metal-based catalysts. To overcome this challenge, researchers have explored various strategies, including doping and hybridization, to enhance the catalytic performance of CQDs. For example, nitrogen-doped CQDs combined with CeO_2_ have been shown to significantly improve photocatalytic hydrogen production. In one study, this hybrid system achieved a hydrogen evolution rate of 10.5 μmol h^−1^, representing a 40% increase compared to pure CQDs, which produced only 7.5 μmol h^−1^.^136^ This improvement is attributed to the enhanced charge separation and electron transfer facilitated by nitrogen doping and the synergistic effects of the CeO_2_ support.

Hybrid CQD catalysts have also demonstrated significant improvements in the ORR and OER, which are critical for energy storage and conversion technologies such as fuel cells and water-splitting systems. For instance, N-substituted CQDs impregnated with Fe_3_O_4_ nanoparticles exhibited high efficiency and stability in the OER, achieving a turnover frequency (TOF) of 0.48 s^−1^ at an overpotential of 320 mV. This hybrid catalyst maintained excellent stability over 1000 cycles, demonstrating its potential for long-term applications in electrochemical reactions.^137^ Similarly, CQDs decorated with transition metal nanoparticles, such as Fe_3_O_4_, have shown remarkable OER performance, further emphasizing the versatility of hybrid CQD catalysts in enhancing the efficiency and stability of electrochemical processes.^138^ The integration of CQDs into more complex hybrid systems has further broadened their applications and improved their catalytic performance. For example, a nanohybrid catalyst comprising cerium oxide–CQDs/reduced graphene oxide (CeO_2_–CQD/RGO) demonstrated enhanced photocatalytic activity compared to pure CQDs. This composite material facilitated better electron transfer and provided more active sites for reactions, leading to improved performance in hydrogen production. The CeO_2_–CQD/RGO composite exemplifies how hybridization can significantly enhance the overall catalytic efficiency of CQDs, making them more competitive with traditional transition metal catalysts.^139^

## 5. Mechanisms of electrocatalysis in CQDs

### 5.1. Adsorption of reactants

The initial step in any electrocatalytic reaction is the adsorption of reactants onto the catalyst surface. CQDs possess a high specific surface area, enabling effective interaction with a broad range of reactants. The surface of CQDs is modified with various oxygen-containing functional groups, such as –COOH, –OH, and epoxy groups, which are crucial for the adsorption process. These functional groups not only facilitate the physical adsorption of reactants but also establish strong electrostatic interactions, hydrogen bonding, and van der Waals forces, allowing reactants to be selectively bound to the CQD surface. This high density of surface functional groups ensures that a large number of reactants can be adsorbed, which is vital for enhancing the overall electrocatalytic efficiency.^17^ For instance, in the HER, protons from the electrolyte are adsorbed onto these surface functional groups, enabling proximity to active sites and promoting reduction. Similarly, in the ORR, molecular oxygen is adsorbed onto CQDs through interactions with nitrogen or sulfur dopants and vacancies, activating the oxygen molecule and weakening the O=O bond for reduction. During the OER, OH^−^ or water molecules are adsorbed on CQD surfaces, particularly at active sites like edge carbon atoms or dopant sites, facilitating electron extraction and bond breaking for oxygen evolution.^50,64,79^

In the CO_2_RR, CO_2_ molecules adsorb onto the CQD surface *via* interactions with defects or heteroatom dopants (*e.g.*, nitrogen and sulfur), enhancing the binding affinity and activation of CO_2_ molecules, which is essential for converting CO_2_ into useful hydrocarbons or alcohols. The bifunctional capability of CQDs, which allows them to adsorb both O_2_ (for the ORR) and OH^−^/H_2_O (for the OER), is particularly valuable in rechargeable battery systems like Zn–air or Li–air batteries, where they can catalyze both reduction and oxidation reactions. Additionally, in liquid fuel electrooxidation, small organic molecules like methanol or ethanol are efficiently adsorbed on CQD surfaces. The surface functional groups stabilize the adsorption, while defects or dopants provide active sites for the subsequent oxidation of fuels in direct methanol fuel cells (DMFCs) and ethanol fuel cells (DEFCs). This versatility in adsorption and activation of various reactants underscores the potential of CQDs as highly efficient electrocatalysts across a wide range of applications.^96,118^

### 5.2. Electron transfer

Efficient electron transfer is critical for any electrocatalytic process, as it dictates the reaction rate and energy efficiency. The electrical conductivity of CQDs, typically ranging from 10^−4^ to 10^−3^ S cm^−1^, stems from their sp^2^ carbon structure, which facilitates the delocalization of π-electrons. These delocalized electrons, further enhanced by quantum confinement effects, enable rapid and efficient electron transport between the CQD surface and the reactants. The small size of CQDs also contributes to a high surface-to-volume ratio, increasing the availability of delocalized electrons for charge transfer. This enhanced electron mobility is essential for accelerating reaction kinetics and improving the overall efficiency of electrocatalytic processes such as hydrogen evolution or oxygen reduction. For example, in the HER, electrons are transferred from the conductive CQD core to adsorbed H^+^, driving the reduction of H^+^ to form H_2_. The presence of heteroatoms like N or S can further enhance electron transfer by modulating the electronic density at active sites, reducing the overpotential for hydrogen evolution. Similarly, in the ORR, electrons are transferred from the CQD surface to the adsorbed O_2_ molecule, weakening the O=O bond and facilitating its reduction to either H_2_O or H_2_O_2_. Defects in CQDs, such as vacancies or heteroatom dopants, act as electron-rich sites, enhancing the overall electron transfer rate.^18,72^

In the OER, electron extraction occurs when OH^−^ or H_2_O molecules lose electrons to the CQD surface. The ability of CQDs to accommodate charge redistribution ensures efficient electron removal, facilitating the formation of O_2_. The use of metal oxide hybrids (CQD–MO) has been shown to significantly improve electron extraction and stability, especially in alkaline media. For the CO_2_RR, stepwise electron transfers to adsorbed CO_2_ molecules lead to intermediate species like COOH and CO. The rapid electron transfer in CQDs, coupled with their quantum confinement, helps reduce overpotentials and improve product selectivity toward hydrocarbons or alcohols. CQDs also excel in bifunctional catalysis, such as in systems requiring both oxygen reduction and evolution (*e.g.*, metal–air batteries). Their ability to mediate electron transfer in both directions (reduction in the ORR and oxidation in the OER) makes them versatile catalysts for energy conversion devices. Additionally, during the electrooxidation of liquid fuels like methanol or ethanol, electron transfer from the organic fuel to the CQD surface enables complete oxidation to CO_2_. The high conductivity of CQDs ensures efficient electron flow during this process, minimizing energy losses and improving fuel cell efficiency. These properties collectively highlight the pivotal role of CQDs in enhancing electron transfer across a wide range of electrocatalytic applications.^52,80,119^

### 5.3. Intermediate stabilization

The stabilization of reaction intermediates is crucial in lowering energy barriers and improving the rate of electrocatalytic reactions. Reaction intermediates, which are often highly reactive and short-lived, need stabilization to prevent premature recombination or decomposition. CQDs offer various mechanisms for intermediate stabilization through their surface chemistry. Functional groups such as –COOH and –OH provide anchoring sites for intermediates, preventing undesired side reactions, while defects like vacancies and edge sites offer localized electronic states that stabilize intermediates during catalysis. These interactions reduce activation energy and enhance reaction selectivity by controlling the formation and transformation of intermediates. In the HER, CQDs stabilize adsorbed H through strong bonding interactions with surface functional groups, facilitating H_2_ formation. In the ORR, intermediates like O_2_^−^ or H_2_O_2_ require stabilization for further reduction, with nitrogen doping enhancing intermediate stabilization, promoting complete reduction to H_2_O. Similarly, oxygen vacancies or dopants in CQDs stabilize intermediates like OH and O during the OER, lowering overpotentials and improving O_2_ evolution.^61,76^

In the CO_2_RR, intermediates such as COOH and CO are critical for reducing CO_2_ to hydrocarbons or alcohols, and nitrogen or sulfur-doped CQDs effectively stabilize these intermediates, lowering reaction energy barriers and enabling selective conversion to CH_4_ or CH_3_OH. CQDs also function as bifunctional catalysts by stabilizing both ORR and OER intermediates, such as O in the OER and O_2_^−^ in the ORR, allowing efficient performance in reversible electrocatalytic systems. In liquid fuel electrooxidation reactions, intermediates like COH or CHO, formed during methanol or ethanol oxidation, are stabilized by surface functional groups, guiding the reaction toward complete oxidation and improving fuel utilization in fuel cell applications.^93^

### 5.4. Desorption of products

Once the reaction occurs, desorption of products from the catalyst surface is a crucial step in maintaining catalytic efficiency. If product molecules remain bound to the active sites, they can block the adsorption of new reactants, leading to catalyst deactivation and reduced reaction rates. Desorption of products from CQDs is influenced by the nature of their interaction with the surface. Oxygen-containing functional groups on CQDs can facilitate weak interactions with the products, promoting efficient desorption. Additionally, the energetic landscape of the CQD surface, which can be fine-tuned by adjusting functionalization, size, and defects, plays a significant role in determining how easily products are released. Efficient desorption keeps the CQD surface available for further reactions, sustaining catalytic performance over time.^18,53^

For the HER, desorption of H_2_ from the CQD surface is enabled by weak interactions between H_2_ and the carbon structure, preventing deactivation of active sites and maintaining high turnover frequencies. In the ORR, desorption of H_2_O or OH^−^ is critical for continuous oxygen reduction, with surface functional groups lowering desorption energy. For the OER, O_2_ must efficiently desorb after formation to prevent oxygen bubble buildup, which could hinder subsequent reactions. Similarly, in the CO_2_RR, products like methane or methanol must desorb from the CQD surface to prevent catalyst poisoning and maintain activity. In bifunctional catalysis systems requiring both the ORR and OER, efficient desorption of O_2_ from the OER and H_2_O from the ORR is essential for preserving the dual-functionality of CQDs. In liquid fuel electrooxidation, desorption of CO_2_ or other products after methanol or ethanol oxidation is necessary to avoid catalyst poisoning, with CQDs promoting efficient desorption due to their optimized surface chemistry and defect density.^70,82,102^

### 5.5. Defect-mediated catalysis

Nitrogen or sulfur doping and defects such as vacancies significantly enhance the electrocatalytic performance of CQDs. These defects introduce localized electronic states that act as additional active sites, increasing the reactivity of CQDs by boosting the density of reactive sites, thus facilitating faster adsorption of reactants. Heteroatom doping, such as nitrogen or sulfur, alters the electronic structure of CQDs, improving charge transfer and lowering activation energies. For instance, nitrogen doping introduces electron-rich sites that aid in the adsorption of certain reactants, while sulfur doping enhances catalytic activity for reactions like the HER. These defects not only increase the number of active sites but also modify the electronic properties of CQDs, making them more effective in specific electrocatalytic processes.^77^

In the HER, defects such as vacancies and dopants provide additional proton adsorption sites, promoting the breaking of the H–H bond and facilitating the electron transfer needed to reduce H^+^ to H_2_. In the ORR, carbon vacancies and heteroatom dopants (especially nitrogen) improve oxygen adsorption and electron transfer, weakening the O=O bond for more efficient reduction to water or hydrogen peroxide. In the OER, defects increase the binding strength of OH^−^ or H_2_O molecules, enhancing electron extraction and bond breaking, crucial for improving energy efficiency in water splitting. In the CO_2_RR, defects offer active sites that stabilize intermediates like COOH and CO, aiding in the multi-step electron transfer process to convert CO_2_ into hydrocarbons or alcohols. Defects also allow CQDs to serve as bifunctional catalysts in both the ORR and OER, improving oxygen adsorption and reaction efficiency in energy conversion systems like metal–air batteries. Additionally, in liquid fuel electrooxidation, defects enhance the adsorption of small organic molecules such as methanol or ethanol, promoting their electrooxidation into CO_2_, which is critical for fuel cell applications like direct methanol or ethanol fuel cells.^54,92,110^

### 5.6. pH-dependent electrocatalysis

The pH of the electrolyte solution plays a critical role in determining the electrocatalytic behavior of CQDs. It influences the protonation state of surface functional groups, which in turn affects their ability to interact with reactants. Furthermore, pH determines the availability of specific reactant species, influencing the overall reaction mechanism. For example, in acidic environments, protonated species may be more abundant, favoring reactions like hydrogen evolution, while under alkaline conditions, hydroxide ions become more significant for processes such as oxygen reduction. By adjusting the pH of the electrolyte, the electrocatalytic performance of CQDs can be tailored for specific reactions, making them highly versatile for applications like fuel cells, water splitting, and CO_2_ reduction. This pH sensitivity offers a strategic advantage for designing CQD-based catalysts that are adaptable across a wide range of electrochemical reactions.^3,7^

For the HER, acidic media enable CQDs to efficiently catalyze proton reduction, as surface functional groups like –COOH and –OH interact with H^+^, stabilizing transition states and lowering activation energies to enhance hydrogen gas production. In contrast, for the ORR, CQDs exhibit better activity under alkaline conditions, where defects or dopants aid in oxygen adsorption and its reduction to water or hydrogen peroxide, with the alkaline environment stabilizing intermediates and accelerating the reaction rate. Similarly, in the OER, higher pH levels favor the adsorption of OH^−^ or water on CQD surfaces, promoting electron extraction and bond cleavage to form oxygen gas. For the CO_2_RR, alkaline conditions enhance CO_2_ adsorption and its reduction to intermediates like COOH or CO, facilitating electron transfer for conversion to hydrocarbons or alcohols. Additionally, the pH-dependent properties of CQDs allow them to serve as bifunctional catalysts, excelling in the HER under acidic conditions while promoting the ORR and OER in alkaline environments, making them suitable for systems like rechargeable metal–air batteries. Finally, pH impacts the electrooxidation of liquid fuels, where acidic media enhance methanol or ethanol oxidation to CO_2_, facilitated by proton-rich environments on the CQD surface.^57,91,113^

### 5.7. Synergistic effects in hybrid catalysts

CQDs, when combined with materials such as metal nanoparticles, metal oxides, or conducting polymers, exhibit synergistic effects that greatly enhance their electrocatalytic performance. The combination with metals like platinum or gold significantly improves charge transfer efficiency, while metal oxides such as iron oxide or manganese oxide provide additional active sites and improve catalyst stability. These hybrid structures benefit from the complementary properties of both CQDs and other materials—CQDs promote electron transfer and stabilize reaction intermediates, while the metals or metal oxides enhance the active site availability and catalytic robustness. This synergy leads to notable improvements in catalytic efficiency, selectivity, and durability. Furthermore, these hybrid catalysts display superior resistance to degradation, making them particularly well-suited for long-term applications in renewable energy technologies.^53,67^

In the HER, CQD–metal hybrids leverage the conductive nature of CQDs for electron transfer, while metals such as platinum or nickel act as active sites for proton reduction, resulting in lower overpotentials and faster hydrogen production. For the ORR, CQD–metal oxide hybrids work together to enhance efficiency, with metal oxides aiding oxygen adsorption and reduction while CQDs provide stability and conductivity. Similarly, in the OER, CQD–metal oxide hybrids are highly effective, as CQDs improve electron flow and stability while metal oxides facilitate the electron extraction needed for O_2_ generation. In the CO_2_RR, hybrid catalysts enhance CO_2_ adsorption and intermediate stabilization, with metals catalyzing the reduction and CQDs improving overall efficiency and product selectivity. Bifunctional catalysts utilizing CQD–metal hybrids are especially useful in rechargeable energy devices like metal–air batteries, where CQDs act as conductive bridges facilitating both the ORR and OER. Additionally, in fuel cells, CQD hybrids improve the electrooxidation of methanol or ethanol, offering better stability and efficiency for applications in direct methanol fuel cells (DMFCs) and ethanol fuel cells (DEFCs).^76,91,115^

### 5.8. Size-dependent properties

The unique properties of CQDs are heavily influenced by their size, typically ranging from 1 to 10 nm. At this nanoscale, CQDs exhibit a high surface-to-volume ratio, significantly increasing the number of active sites available for reactant adsorption. This characteristic makes them highly effective catalysts for various electrochemical processes. Additionally, their small size leads to pronounced quantum effects, such as quantum confinement, which modify the electronic band structure. This can reduce the activation energy required for reactions, thereby enhancing their catalytic properties. The small size of CQDs also improves charge carrier mobility, allowing for faster electron transport. These size-dependent properties make CQDs highly tunable, enabling the optimization of their electrocatalytic performance for a wide range of applications, including energy conversion and storage, sensing, and environmental remediation.^2,5^

In the HER, the small size of CQDs ensures a greater number of active sites for proton adsorption, enhancing hydrogen gas production. For the ORR, the high surface area and nanoscale size improve the mass transport of oxygen molecules to the active sites, increasing ORR efficiency. Similarly, for the OER, the small size of CQDs facilitates efficient electron extraction, promoting the bond-breaking steps required for oxygen formation. In the CO_2_RR, the nanoscale dimensions enhance CO_2_ adsorption and the formation of reaction intermediates, reducing overpotentials and improving the process. The high density of active sites also makes CQDs suitable for bifunctional catalysts, where both the ORR and OER are required. In liquid fuel electrooxidation, the small size of CQDs enhances the adsorption and oxidation of fuels like methanol or ethanol, resulting in higher fuel cell performance with minimal energy loss.^70,90,112^Table 2 is prepared based on the references cited in the text, displaying the electrocatalytic mechanisms and performance of CQDs.

**Table 2:** Electrocatalytic mechanisms and performance of CQDs

Mechanism	Description	Key applications
Adsorption of reactants	CQDs possess a high specific surface area (∼100–700 m^2^ g^−1^), allowing efficient reactant adsorption. Functional groups (–COOH, –OH, and –NH_2_) and defects facilitate adsorption through electrostatic interactions (∼50–200 meV), hydrogen bonding (∼10–50 kJ mol^−1^), van der Waals forces (∼0.4–4 kJ mol^−1^), and π–π interactions (∼2–8 kJ mol^−1^)	HER (H^+^ adsorption), ORR (O_2_ adsorption *via* dopants), CO_2_RR (CO_2_ adsorption *via* defects), OER (OH^−^/H_2_O adsorption), and bifunctional catalysts (O_2_ & OH^−^/H_2_O adsorption in batteries)
Electron transfer	CQDs exhibit electrical conductivity in the range of 10^−4^ to 10^−2^ S cm^−1^, depending on synthesis conditions and doping. Their sp^2^ hybridized structure facilitates rapid electron transfer (∼10^5^ to 10^6^ cm^2^ V^−1^ s^−1^ in well-structured CQDs). Quantum confinement alters the bandgap (typically 1.5–3.2 eV), influencing charge transport and reaction kinetics	HER (H^+^ reduction), ORR (O_2_ reduction), CO_2_RR (CO_2_ conversion), OER (O_2_ evolution), and liquid fuel electrooxidation (methanol/ethanol oxidation)
Intermediate stabilization	Functional groups (–COOH, –OH, quinones, and epoxides) and defects (vacancies and heteroatoms) stabilize reaction intermediates, reducing activation energy by 0.2–0.6 eV in the HER, ORR, and CO_2_RR. This stabilization enhances selectivity, with CO_2_RR intermediates (*e.g.*, COOH and CO) achieving binding energies from −0.5 to −1.1 eV on doped CQDs	HER (H stabilization), ORR (O_2_^−^/H_2_O_2_ stabilization), CO_2_RR (COOH/CO stabilization), OER (OH/O stabilization), and liquid fuel electrooxidation (COH/CHO stabilization)
Desorption of products	Weak product–catalyst interactions (desorption energies of ∼0.2–0.5 eV) prevent deactivation. Controlled desorption modulates selectivity, with methane or methanol in the CO_2_RR requiring intermediate adsorption energies (−0.3 to −0.8 eV) for optimal performance	HER (H_2_ desorption), ORR (H_2_O/OH^−^ desorption), CO_2_RR (controlled CH_4_/CH_3_OH desorption), OER (O_2_ desorption), and bifunctional catalysts (O_2_ & H_2_O desorption)
Defect-mediated catalysis	Defects, such as nitrogen doping or vacancies, introduce localized charge redistribution (∼0.1–0.4 eV shift in density of states), enhancing reactant adsorption and catalytic activity. ORR activity is improved with N-doped CQDs, achieving an onset potential of ∼0.85–0.90 V *vs.* RHE	HER (proton adsorption sites), ORR (N-doped oxygen adsorption), CO_2_RR (COOH/CO stabilization), OER (OH^−^/H_2_O binding), and liquid fuel electrooxidation (methanol/ethanol oxidation)
pH-dependent electrocatalysis	CQDs adapt to pH variations, influencing surface charge and reaction pathways. In acidic HER (pH 0–3), a high proton concentration enables faster kinetics (∼−0.1 to −0.2 V overpotential). The ORR in alkaline media (pH 13–14) follows a four-electron pathway, reducing O_2_ with a half-wave potential of ∼0.80 V *vs.* RHE. The CO_2_RR under alkaline conditions improves CO_2_ adsorption, lowering overpotentials to ∼0.3–0.6 V	HER (acidic media for H_2_), ORR (alkaline O_2_ reduction), OER (high pH O_2_ evolution), CO_2_RR (alkaline CO_2_ reduction favoring specific products), and bifunctional catalysts (HER in acid and ORR/OER in alkaline media)
Synergistic effects in hybrid catalysts	Hybrid CQDs (*e.g.*, with Pt, Au, and Fe_2_O_3_) enhance catalytic efficiency by lowering overpotentials (by ∼50–120 mV) and improving current densities (up to 10–100 mA cm^−2^, depending on the system). Charge transfer at CQD–metal interfaces (Schottky barriers ∼0.1–0.3 eV) improves reaction kinetics	HER (CQD–metal hybrids like Pt/Ni), ORR (CQD–metal oxides), OER (electron extraction), CO_2_RR (CO_2_ adsorption/selectivity), bifunctional catalysts (ORR/OER in batteries), and liquid fuel electrooxidation (enhanced methanol/ethanol oxidation)
Size-dependent properties	CQDs (1–10 nm) exhibit a high surface-to-volume ratio, increasing active site density. Quantum confinement increases the bandgap (1.5–3.2 eV for smaller CQDs), enhancing catalytic efficiency. Small CQDs also increase defect density (∼10^12^ to 10^13^ cm^−2^), further boosting performance	HER (increased active sites), ORR (better O_2_ transport), OER (efficient electron extraction), CO_2_RR (enhanced adsorption), bifunctional catalysts (efficient ORR/OER), and liquid fuel electrooxidation (improved methanol/ethanol oxidation)

## 6. Challenges, advancements, and future potential of electrocatalytic behavior of CQDs

### 6.1. Challenges in electrocatalytic applications of CQDs

CQDs have attracted significant attention in electrocatalysis due to their tunable physicochemical properties, high surface area, and eco-friendly synthesis. However, their application in key electrochemical reactions such as the OER, HER, ORR, CO_2_RR, and liquid fuel electrooxidation faces several critical challenges that hinder their widespread use. These challenges include intrinsic conductivity issues, structural instability, limited active sites, scalability concerns, and selectivity in multi-electron transfer reactions. Addressing these limitations is essential for unlocking the full potential of CQDs in sustainable energy conversion and storage systems.

One of the primary limitations of CQDs is their inherently low electrical conductivity, which restricts efficient charge transfer during electrocatalytic reactions. Since CQDs are primarily composed of sp^2^ and sp^3^ hybridized carbon, their electronic properties do not match those of highly conductive materials like graphene or metal-based catalysts. This challenge is particularly problematic in reactions such as the OER and ORR, where rapid electron transfer is crucial for efficient catalytic performance. To enhance their conductivity, researchers have explored hybridization with conductive materials such as GO, reduced GO, and transition metal oxides.^122^ However, achieving a uniform and stable hybrid structure remains a challenge. CQDs tend to aggregate due to their high surface energy, leading to a reduction in available active sites for catalytic reactions. This issue is especially significant in the HER and OER, where a well-dispersed catalyst surface is required for optimal interaction with the electrolyte. The aggregation of CQDs can result in uneven charge distribution, poor mass transport, and diminished catalytic performance. Additionally, structural instability under operational conditions can lead to loss of catalytic activity over time. Strategies such as surface modification, polymer encapsulation, and functionalization with stabilizing agents have been explored to mitigate aggregation, but further improvements are required for practical applications.^62^

The long-term stability of CQDs in harsh electrochemical environments remains a critical concern. Under highly acidic or alkaline conditions, commonly used in water-splitting and fuel cell applications, CQDs may undergo oxidation, degradation, or structural reconstruction, leading to performance degradation. This challenge is particularly relevant in the OER and ORR, where prolonged cycling can lead to carbon corrosion and catalyst deactivation. To address this, researchers have focused on developing heteroatom-doped CQDs and hybrid composites with transition metal oxides, phosphides, or sulfides to improve stability.^51^ However, further research is needed to understand degradation mechanisms and enhance the robustness of CQDs under operational conditions. Surface functional groups play a crucial role in determining the electrocatalytic activity of CQDs by modulating their electronic structure and active site availability. However, these functional groups often degrade during prolonged electrocatalysis, reducing their effectiveness in reactions such as the HER and CO_2_RR. For example, oxygen-containing functional groups can enhance catalytic activity by facilitating charge transfer, but they may be leached out under strong electrochemical cycling.^54^ Ensuring the stability of functionalized CQDs requires advanced synthetic strategies such as covalent bonding of functional groups or protective coatings to prevent degradation.

Despite their high surface area, CQDs often exhibit sluggish charge transfer kinetics, which can limit their performance in multi-electron transfer reactions such as the ORR and CO_2_RR. The sluggish kinetics arises from the limited density of active sites and the non-metallic nature of CQDs, which restricts their ability to facilitate rapid electron transfer. To overcome this, researchers have investigated heteroatom doping, wherein elements such as nitrogen, sulfur, or phosphorus introduce localized charge redistribution, enhancing charge transport properties.^79^ Additionally, transition metal incorporation into CQD frameworks has shown promise in boosting charge transfer efficiency, but achieving a precise balance between charge transfer and catalytic activity remains a challenge. For bifunctional electrocatalysts, such as those designed for the OER/ORR or HER/CO_2_RR, CQDs require precise engineering to balance oxidation and reduction activities. However, CQDs often have a limited number of active sites, which restricts their catalytic efficiency in dual-function applications. This challenge is particularly relevant in rechargeable metal–air batteries and water-splitting applications, where a single material must exhibit both excellent oxygen evolution and reduction capabilities. Strategies such as dual-heteroatom doping, hybridization with transition metals, and defect engineering have been explored to improve bifunctional performance.^130^ However, these approaches require further optimization to achieve practical, high-performance bifunctional electrocatalysts.

A major bottleneck in the commercialization of CQD-based electrocatalysts is the challenge of producing high-quality CQDs at an industrial scale with consistent size, morphology, and surface properties. Traditional synthesis methods, including hydrothermal, solvothermal, and electrochemical routes, often result in CQDs with broad size distributions and varying functionalization. This inconsistency affects their catalytic performance and reproducibility. Although green synthesis methods using biomass-derived CQDs have been proposed as a cost-effective and scalable alternative, achieving precise control over their physicochemical properties remains difficult.^49^ In CO_2_ reduction, achieving selectivity for specific products such as CO, CH_4_, or methanol is challenging due to competing reaction pathways. CQDs alone often show low selectivity due to their limited ability to stabilize reaction intermediates. This problem is compounded by their non-metallic nature, which lacks the electronic states required to guide specific reaction mechanisms. To improve selectivity, researchers have explored strategies such as co-catalyst integration, heteroatom doping, and defect engineering to modify the electronic structure of CQDs. However, fine-tuning these properties to achieve high selectivity without compromising catalytic efficiency remains a significant challenge.^102^

### 6.2. Advanced strategies for enhancing CQD electrocatalysts

Doping CQDs with heteroatoms such as N, S, P, and B significantly enhances their electrocatalytic properties by altering the electronic structure, introducing localized charge redistribution, and increasing active sites for catalytic reactions. Nitrogen doping enhances electron-donating properties, improving catalytic activity in the HER and ORR.^53^ Sulfur and phosphorus doping modulates charge density and stabilizes reaction intermediates in the OER.^55^ Additionally, dual-heteroatom doping, such as N,S or N,P doping, creates synergistic effects that enhance charge transfer efficiency and durability. To improve conductivity and catalytic efficiency, CQDs have been integrated with transition metal-based catalysts such as metal oxides, perovskites, phosphides, sulfides, and nitrides, enhancing charge transfer kinetics and introducing additional active sites. CQD–metal oxide hybrids, including Fe_2_O_3_ and Co_3_O_4_, have shown improved ORR and OER activity.^58^ Incorporation into materials like Ni_2_P and MoS_2_ has enhanced HER performance by lowering the energy barrier for hydrogen evolution. Furthermore, CQDs have been used to improve the catalytic activity of perovskite-based materials in the OER and CO_2_RR.^91^

Electrodeposition techniques have been developed to integrate CQDs with transition metal-based catalysts in a single step, ensuring uniform dispersion and improved catalytic stability. This method has been particularly effective in HER and OER applications, where homogeneous catalyst distribution enhances overall efficiency.^54^ Additionally, electrodeposited CQDs have demonstrated enhanced conductivity, making them promising candidates for fuel cell applications. To address the issue of electrochemical durability, CQDs have been encapsulated within protective layers such as reduced GO (rGO), polymers, or metal–organic frameworks (MOFs). These encapsulation strategies help prevent degradation of functional groups and maintain catalytic efficiency over prolonged operation. rGO enhances the conductivity and stability of CQDs in the OER and ORR.^58^ Functional polymer coatings improve resistance against acidic and alkaline degradation, extending the lifetime of CQD-based catalysts.^54^ Metal–organic frameworks (MOFs) provide a high surface area and structural stability, improving the catalytic performance of CQDs in the CO_2_RR.^102^

To enable large-scale applications, researchers have explored environmentally friendly synthesis routes for CQDs. Biomass-derived CQDs produced through green synthesis methods using natural precursors such as plant extracts and waste biomass have shown enhanced electrocatalytic properties.^139^ Additionally, hydrothermal and microwave-assisted synthesis methods offer precise control over CQD size and surface functionalization, improving their catalytic efficiency in the HER and OER.^107^ Introducing structural defects in CQDs has been shown to enhance their catalytic performance by increasing active site density and improving charge transfer kinetics. Oxygen vacancy-rich CQDs, created through defect engineering strategies, have demonstrated improved OER activity.^110^ Similarly, defective carbon structures facilitate rapid electron transport, boosting catalytic efficiency in ORR applications.^91^ CQDs have been optimized for dual-function applications, particularly in OER/ORR and HER/CO_2_RR systems. In water-splitting applications, CQDs have been used in integrated systems to catalyze both the HER and OER, improving overall efficiency.^60^ Additionally, CQD-based bifunctional electrodes have been explored for use in rechargeable zinc–air and lithium–air batteries, enhancing both energy density and cycling stability.^133^

CQDs have been explored as supporting materials for metal-based catalysts in direct methanol and ethanol fuel cells, significantly improving fuel oxidation efficiency. CQD-supported platinum and palladium nanocatalysts have demonstrated improved electrochemical activity in methanol oxidation reactions, offering a potential alternative to conventional Pt-based catalysts.^117^ Similarly, CQD-based nickel and cobalt electrocatalysts have exhibited high catalytic performance in ethanol oxidation, making them promising for sustainable fuel cell applications.^62^ The conversion of CO_2_ into valuable fuels and chemicals using CQD-based electrocatalysts is a promising strategy for mitigating climate change while producing sustainable energy carriers. However, selectivity remains a challenge due to competing reaction pathways. Engineering CQDs with controlled surface functionalization and electronic properties can enhance CO_2_-to-CO selectivity, which is valuable for syngas production.^99^ Integration of CQDs with transition metals such as Cu and Fe has shown improved CO_2_ reduction efficiency, reducing the overpotential and increasing the product yield. Additionally, combining CQDs with semiconductor photocatalysts enables solar-driven CO_2_ reduction, offering a sustainable approach to carbon recycling and fuel generation.^128^ Significant advancements in CQD-based electrocatalysts have been achieved through heteroatom doping, hybridization with transition metals, defect engineering, and encapsulation strategies. These improvements have enhanced charge transfer efficiency, catalytic activity, and long-term stability in reactions such as the OER, the HER, the ORR, the CO_2_RR, and liquid fuel electrooxidation. Additionally, scalable and green synthesis methods have paved the way for the commercialization of CQD-based catalysts in sustainable energy applications. However, further research is needed to optimize bifunctional performance, improve selectivity in the CO_2_RR, and develop cost-effective large-scale production techniques.

### 6.3. Future potential of CQD-based electrocatalysts

The rapid advancements in CQD-based electrocatalysts have opened up new frontiers in energy storage and conversion technologies. CQDs offer unique advantages such as high surface area, tunable electronic properties, excellent conductivity, and superior electrocatalytic activity, making them promising candidates for various electrochemical applications. Future research and technological developments are expected to further improve their performance, scalability, and integration into commercial energy systems. This section explores the potential applications of CQD-based electrocatalysts and their role in advancing sustainable energy solutions. Metal-free electrocatalysts are highly desirable due to their cost-effectiveness, abundance, and environmental sustainability. CQDs are being investigated as metal-free alternatives for fuel cells, the HER, and the ORR, potentially replacing expensive platinum-based catalysts.^63^ Nitrogen-doped CQDs (N-CQDs) have demonstrated remarkable ORR performance by providing active sites for oxygen adsorption and facilitating efficient electron transfer.^78^ Additionally, phosphorus and sulfur co-doped CQDs have shown promising HER activity by modulating the electronic structure and charge density.^61^ As research advances, CQDs could be optimized to serve as efficient and durable metal-free catalysts for a wide range of electrochemical reactions.

The fluorescence and electrochemical properties of CQDs enable their application in multifunctional electrochemical sensors. CQD-based smart sensors can be used for self-powered electrochemical sensing, environmental monitoring, and real-time biomolecule detection.^137^ These sensors leverage the high conductivity and large surface area of CQDs to enhance sensitivity and selectivity. For example, nitrogen and sulfur co-doped CQDs have been employed for detecting heavy metal ions, providing high sensitivity and stability in complex matrices.^59^ Moreover, CQDs functionalized with biomolecules enable selective recognition of glucose, dopamine, and other analytes, paving the way for the development of next-generation biosensors with real-time detection capabilities. The integration of CQDs with two-dimensional (2D) materials such as black phosphorus, MXenes, and layered hydroxides offers new opportunities for enhancing catalytic efficiency and selectivity in the CO_2_RR and ORR.^57^ CQD/MXene hybrids exhibit excellent charge transfer kinetics and superior electrochemical stability, making them suitable for electrocatalytic applications. Additionally, CQD-functionalized black phosphorus has demonstrated remarkable performance in the CO_2_RR by improving electron-donating capabilities and facilitating CO_2_ adsorption.^95^ These hybrid nanomaterials provide synergistic effects that enhance catalytic activity, durability, and selectivity, making them attractive candidates for energy conversion processes.

Water electrolysis is a key process for green hydrogen production, and CQD-based electrocatalysts are emerging as promising alternatives to conventional catalysts. The development of scalable synthesis techniques, such as hydrothermal and microwave-assisted methods, has enabled the large-scale production of CQD-based catalysts with high electrocatalytic efficiency. CQDs have been successfully integrated with transition metal oxides and perovskites to enhance OER activity, demonstrating potential for industrial-scale application.^52^ Furthermore, CQD-modified nickel and cobalt hydroxides have shown excellent stability and performance in alkaline water electrolysis, providing a cost-effective and sustainable approach for hydrogen generation. Bifunctional catalysts capable of efficiently catalyzing both the OER and HER are essential for rechargeable metal–air batteries and overall water splitting systems. CQD-based bifunctional catalysts have been explored for applications in zinc–air and lithium–air batteries, offering enhanced energy density and cycling stability.^60^ CQD-functionalized NiCo layered double hydroxides have exhibited superior performance in both the HER and OER, demonstrating their potential for energy storage applications.^67^ The combination of CQDs with metal–organic frameworks (MOFs) has also been reported to improve electrocatalytic performance, providing a high surface area and stable active sites for efficient charge transfer. As research progresses, optimizing CQD-based bifunctional catalysts could lead to more efficient and scalable energy storage solutions.

The use of CQDs in electrochemical carbon capture and CO_2_ reduction is gaining significant attention due to their ability to modulate the electronic structure and enhance selectivity. CQDs have been employed to improve the efficiency of CO_2_-to-CO conversion, which is valuable for syngas production.^102^ Additionally, CQD–metal hybrid catalysts, such as CQD-modified Cu and Fe catalysts, have demonstrated enhanced CO_2_ reduction activity by lowering overpotentials and increasing the product yield. CQD-based photocatalytic systems are also being explored for solar-driven CO_2_ conversion, offering a sustainable approach to carbon recycling and fuel generation.^128^ CQD-supported catalysts are being developed to improve the efficiency of methanol, ethanol, and formic acid oxidation in fuel cells. These catalysts play a crucial role in the development of high-performance energy devices for portable and stationary power applications. CQD-supported platinum and palladium nanocatalysts have demonstrated improved electrochemical activity in methanol oxidation, offering a potential alternative to conventional Pt-based catalysts.^117^ Similarly, CQD-based nickel and cobalt electrocatalysts have exhibited high catalytic performance in ethanol oxidation, making them promising for sustainable fuel cell applications.^52^ The ability of CQDs to enhance charge transfer kinetics and provide additional active sites makes them ideal for improving liquid fuel oxidation reactions. The continuous advancements in heteroatom doping, hybrid nanostructures, green synthesis, and defect engineering are driving CQD-based electrocatalysts toward real-world applications. Their potential in sustainable energy technologies, particularly in hydrogen production, CO_2_ reduction, and fuel cells, highlights their importance as next-generation electrocatalytic materials. By addressing existing challenges and leveraging recent developments in nanotechnology, CQD-based electrocatalysts could play a crucial role in the transition toward cleaner and more efficient energy systems.

## 7. Conclusion

CQDs have solidified their status as versatile nanomaterials in electrocatalysis, driven by their exceptional optical, electrical, and catalytic properties. This review has demonstrated their efficacy across pivotal energy conversion reactions, including the OER, HER, ORR, CO_2_RR, and liquid fuel electrooxidation, while also showcasing their promise as bifunctional catalysts. Unlike earlier studies, we have underscored recent breakthroughs in synthesis techniques—such as heteroatom doping, defect engineering, and hybridization with transition metals—that address challenges like low conductivity and structural instability, enhancing their catalytic efficiency and stability for sustainable energy systems. Looking forward, the prospects of CQDs are highly promising, with transformative potential in several key areas. These include their development as metal-free electrocatalysts for cost-effective and environmentally friendly energy conversion, their integration into smart electrochemical sensors for real-time environmental monitoring, and their scalability for industrial green hydrogen production. Future research should prioritize overcoming the remaining hurdles, such as optimizing large-scale synthesis methods for consistent quality and yield, advancing precise functionalization to tailor their electronic properties, and exploring their compatibility with emerging technologies like artificial photosynthesis and carbon capture systems. By addressing these challenges, CQDs are poised to become cornerstone materials in the global transition to eco-friendly and sustainable energy solutions.

## Author contributions

F. F. Sead and H. Noorizadeh conceptualized the study. F. F. Sead led the systematic literature review on structural innovations (*e.g.*, heteroatom doping and defect engineering) and drafting the sections on optical/electrical properties. Yashwantsinh Jadeja analyzed electrocatalytic mechanisms (OER, HER, and ORR) and designed mechanistic schemes, while Anjan Kumar contributed critical insights into the CO_2_RR and bifunctional catalysis, linking structural properties to performance. Rekha M. M. curated advancements in CQD synthesis methods and characterization techniques, emphasizing scalability challenges. Mayank Kundlas and Suman Saini compiled comparative data on catalytic efficiency and assisted in editing, with Kamal Kant Joshi integrating environmental applications and sustainability perspectives. Hadi Noorizadeh, as the corresponding author, coordinated interdisciplinary linkages (*e.g.*, transition metal hybridization), supervised revisions, and finalized the manuscript. All authors collaboratively addressed knowledge gaps, reviewed emerging trends, and approved the final version.

## Conflicts of interest

The authors declare no conflicts of interest.

## Data Availability

No new data were created or analyzed in this study. This review article is based on previously published research and datasets, which are cited and referenced throughout the manuscript. All data sources used in this work are publicly available and can be accessed through the references provided.
